# Knowledge, attitudes, and practices among livestock owners, traders, and slaughterhouse inspectors in Cameroon reveals marginal understanding of livestock and human brucellosis

**DOI:** 10.3389/fvets.2025.1677520

**Published:** 2026-01-30

**Authors:** Charles Olivier Gomsu Dada, Christopher G. Laine, Gaelle Kamdjo Guela, Pierre Gontao, Daniel Garcia-Gonzalez, Mohamed Moctar Mouliom Mouiche, Abel Wade, Angela M. Arenas-Gamboa

**Affiliations:** 1 National Veterinary Laboratory (LANAVET), Garoua, Cameroon; 2 Department of Veterinary Pathobiology, College of Veterinary Medicine and Biomedical Sciences, Texas A&M University, College Station, TX, United States; 3 School of Veterinary Medicine and Sciences, University of Ngaoundéré, Ngaoundéré, Cameroon

**Keywords:** brucellosis, *Brucella*, brucellosis KAP, brucellosis epidemiology, Cameroon

## Abstract

**Introduction:**

Brucellosis, a zoonotic disease, significantly impacts animal and public health, as well as agricultural economies reliant on livestock. This disease is endemic in many regions worldwide, with the highest risk of infection in sub-Saharan Africa. However, the true extent of the disease in Africa remains largely unknown. In Cameroon, a country in western Central Africa, *Brucella abortus* is endemic in livestock, and the bacteria have been found in milk sold at community markets throughout the country.

**Methods:**

This study aims to understand the knowledge, attitudes, and biosecurity practices of those closely working with animals throughout the livestock supply chain from farm to slaughter. Three KAP surveys were conducted among livestock owners, traders, and slaughter facility inspectors at live markets and abattoirs in the Far North, North, and West regions of the country due to their pivotal importance in the national and international supply chain.

**Results:**

Findings reveal minimal understanding of brucellosis and limited biosecurity practices across the network of owners, traders, and abattoir inspectors.

**Discussion:**

The lack of understanding and deficiencies in health infrastructure likely contribute to the persistence of *B. abortus* as endemic in the country and region as a whole. This study provides insights into animal and public health risks and aims to aid policymakers in developing interventions to reduce the disease burden.

## 1 Introduction

Brucellosis, a disease caused by Gram-negative bacteria from the genus *Brucella*, is particularly virulent to both animals and humans. Three of the zoonotic *Brucella* species predominantly affect specific livestock: *Brucella abortus* in cattle, *Brucella melitensis* in sheep and goats, and *Brucella suis* in swine (1–3). This disease significantly impacts animal and public health, as well as agricultural economies reliant on these livestock (1, 3). Particularly, in humans, brucellosis is a major global health concern, with an estimated 2.1 million (potentially 7.45 million) new cases annually (4, 5).

In animals, transmission commonly occurs by way of aerosolization, particularly when they contact contaminated reproductive waste, such as uterine secretions, fetal membranes, placenta, or aborted fetuses while commingling with infected animals, which often leads to abortion, weak offspring, infertility, and decreased milk production (3, 6). Human infection primarily occurs while handling reproductive waste, putting animal handlers, abattoir workers, and veterinarians at substantial risk in areas where the disease is endemic (1, 3). Additionally, people can be exposed through the consumption of unpasteurized milk products, magnifying risk into the community (1, 3). Clinically, similar to malaria, brucellosis normally manifests with non-specific flu-like symptoms such as fever, fatigue, sweats, malaise, and arthritis, and when left untreated, could lead to severe conditions like endocarditis and neurological disorders (3, 7).

Brucellosis is endemic in many regions globally, with the highest number of cases likely occurring in sub-Saharan Africa (4, 5, 8). Although this population faces the greatest burden of disease, the true scope and impact are largely unknown due to several challenges. These include insufficient or non-existent diagnostic capabilities, surveillance, and control systems, as well as limited awareness among veterinary and human health professionals (9–11). However, recent studies have identified the western Central African country of Cameroon as endemic with *B. abortus* in livestock, with bacteria detected in milk sold at open community markets across the country (12, 13). Additionally, the *B. abortus* strain has been epidemiologically linked throughout the country, originating from Sudan and Uganda, likely transmitted along the livestock supply chain traversing sub-Saharan Africa (12–14). However, there remains a systemic absence of livestock traceability, poor national disease surveillance, and inadequate control measures in the country and across borders.

Given that brucellosis is recognized as an occupational disease and biosecurity practices are crucial for its control and prevention, this study aims to understand the knowledge of brucellosis, attitudes toward infectious diseases, and biosecurity practices of animal owners and those closely working with animals, in order to identify areas from farm to slaughter needing targeted interventions. Specifically, in this study, we describe the results obtained from three knowledge, attitude, and practice (KAP) surveys conducted among (1) livestock owners, (2) livestock traders, and (3) slaughter facility inspectors. Our findings reveal that: (1) individuals working with animals have a limited understanding of brucellosis in both animals and humans, and (2) current biosecurity practices at farms, markets, and slaughter facilities are minimal to nonexistent. The lack of understanding throughout the entire livestock supply chain, combined with deficiencies in livestock and public health infrastructure are likely contributing factors to the persistence of brucellosis in the country. It is our expectation that the evidence gathered in this study will offer a clearer insight into the animal and public health risks from farm to slaughter and will ultimately help aid policymakers in developing new interventions to reduce the disease burden.

## 2 Materials and methods

### 2.1 Study design

**Figure 1:**
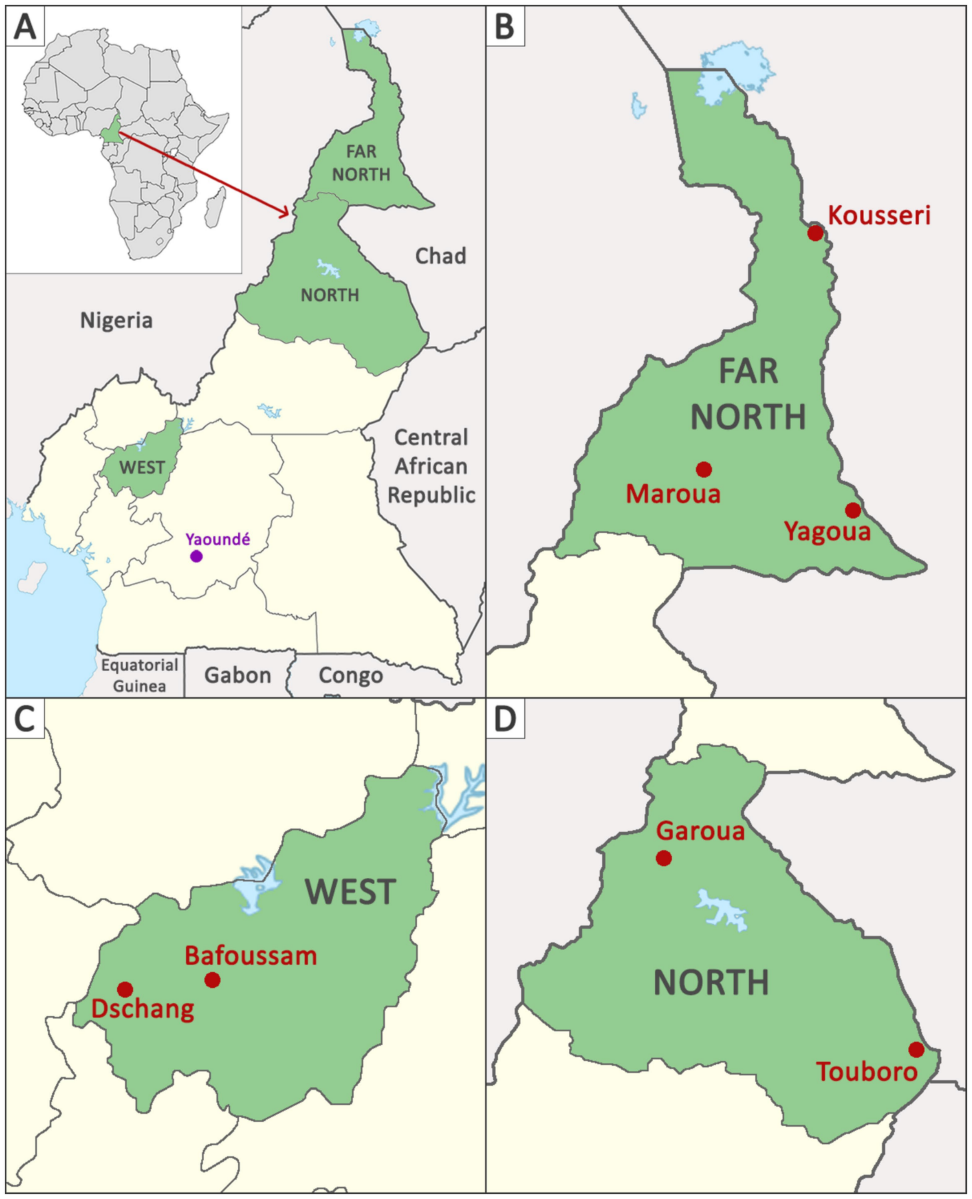
Map of Cameroon. Sampling regions of Cameroon **(A)**, including the Far North **(B)**, West **(C)**, and North **(D)** regions shaded in green, along with the sample site cities (Kousseri, Maroua, Yagoua, Dschang, Bafoussam, Garoua, and Touboro) in red. The location of laboratory analysis (Yaoundé) is depicted in purple.

From February 2021 to May 2023, KAP surveys were administered to randomly selected (1) livestock owners, (2) livestock traders at slaughter facilities and livestock markets, and (3) governmental meat inspectors at specific slaughter facilities in each of the three study regions of Cameroon: the Far North, North, and West. In the Far North, surveys were collected from the cities of Maroua, Yagoua, and Kousseri (Figure 1). In the North, Garoua and Touboro were chosen as survey locations, while in the West, Bafoussam and Dschang were included. These sites were chosen based on studies indicating that *B. abortus* is endemic in the country, impacting both livestock and people at these sample locations (12, 13).

Three structured, closed-ended surveys were employed to gather information on demographics, livestock ownership and management, knowledge of brucellosis, attitudes and practices toward disease control and prevention, and biosecurity practices. The survey questions and answers are available in the Supplementary Tables 1–3.

At each survey site, a local researcher recruited a participant and then administered the respective survey in the preferred language (English or French) using an electronic tablet with Open Data Kit (ODK) Collect software for answer collection and data transfer. Prior to deployment, all three surveys were piloted with five workers in each sample region to ensure appropriate translation between English and French, question clarity and cultural relevance, and proper functioning of the electronic hardware and software.

To achieve the desired statistical power, at least 385 KAP surveys were required per region for each livestock owner and trader population, aiming for a target precision of ±5.0% and a 95% confidence interval (CI), totaling 2,310 surveys across the country. All (100%) of the governmental livestock inspectors at each slaughter facility sample site were surveyed.

### 2.2 Data analysis

Data from each survey population were summarized both nationally and regionally. Proportions were estimated using binomial exact calculations with a 95% confidence interval (CI), weighted by population size. A two-sample *Z*-test of proportions was used to determine significant differences between populations, with positivity rates assessed at a 95% CI and 80% power, and statistical significance defined as *p* ≤ 0.05. When no significant differences were found between regions, data are reported at the national level. When significant differences were observed, data are reported at both the national and regional levels, along with the associated level of significance. In addition, a multivariate analysis was conducted to explore potential associations between demographic and occupational variables (e.g., education level, region, livestock type) and knowledge or biosecurity practices. However, no strong or moderate associations were identified. All analyses were performed using Stata and GraphPad Prism software.

### 2.3 Ethical compliance

This study was evaluated and approved by: (1) the LANAVET research directorate, (2) Texas A&M University (TAMU), Offices of Research Compliance and Biosafety, Human Research Protection Program (HRPP) and United States Department of Defense (DOD), Office of Human Research Oversight (OHRO), Research Oversight Board (ROB) (IRB2020-0974D); and (3) United States Department of Defense (DOD), Defense Threat Reduction Agency (DTRA), Research Oversight Board (ROB) (registration number CT00008). The U. S. Army Medical Research and Development Command, Office of Research Protections (MRDC ORP), Human Research Protections Office (HRPO) registered this effort as E02473 and approved research involving human subjects. The HRPO log numbers are the following: E02473.1a (TAMU Site), E02473.1b-1 (LANAVET-West Region Site), E02473.1b-2 (LANAVET-Far-North Region Site) and E02473.1b-3 (LANAVET-North Region). LANAVET Cameroon deferred to the TAMU IRB and DOD ROB. To ensure best reporting practices, this study was conducted under the Guidelines for Accurate and Transparent Health Estimates Reporting (GATHER) (15).

## 3 Results

Structured, closed-ended KAP surveys were conducted with randomly selected participants from three groups: (1) livestock owners and (2) livestock traders at slaughter facilities and livestock markets, and (3) governmental meat inspectors at specific slaughter facilities. These surveys were carried out in three study regions of Cameroon: the Far North, North, and West. If no significant differences were found between regions, the data are reported at the national level. When significant differences were detected, the data are presented at both the national and regional levels, including the associated level of significance.

Surveys of livestock owners included a total of 1,230 farmers from open-air community slaughter facilities and live markets, with 455 in the Far North, 396 in the North, and 379 in the West (Supplementary Table 1). Livestock traders included a total of 1,147 merchants from open-air community slaughter facilities and live markets, with 385 in the Far North, 374 in the North, and 388 in the West (Supplementary Table 2). All (100%) of the government meat inspectors (36) were included from the open-air community slaughter facilities where livestock owners and traders were surveyed, with 10 in the Far North, 12 in the North, and 14 in the West (Supplementary Table 3).

### 3.1 Livestock owner survey

To begin the farm to slaughter assessment, a survey was conducted among livestock owners at slaughter facilities and livestock markets, with the results detailed in Supplementary Table 1. Demographic information to identify groups that could be targeted for education and intervention was collected throughout the study. Results indicate that livestock owners are predominantly male (1,181; 96.0%) and mostly fall within the age range of 38 to 57 years (990; 80.5%). A substantial portion of the owners are illiterate (687; 56.6%), and 582 (47.5%) have no formal education. Most are small-scale livestock farmers (1,143; 97.1%), with five or fewer individuals caring for the animals, primarily raising goats (825; 70.0%), followed by sheep (755; 64.1%), cattle (643; 54.6%), and pigs (176; 14.9%). Besides livestock ownership, many participants also engage in other income-generating activities, such as crop farming (684; 57.1%) and dairy farming (231; 19.3%).

A review of the farmers’ livestock trade was carried out to identify the types of animals they typically manage and the potential destinations for the livestock once they leave the farm. Although, sharing, lending, or borrowing animals with other farmers is uncommon (125; 10.6%), those who do mainly supply male cattle (65; 52.0%) and male sheep (58; 46.4%). When acquiring new livestock and integrating them into a herd, farmers primarily purchase animals as needed (514; 43.8%) or on a monthly basis (402; 34.2%). When selling, 561 (47.8%) of the respondents sold their live animals for purposes other than slaughter, with 384 (58.1%) selling cattle, 354 (63.2%) selling sheep, 320 (57.1%) selling goats, and only 62 (11.1%) selling pigs. Additionally, despite a large proportion of farmers selling live animals for purposes other than slaughter, 326 (58.1%) sell to butchers (individuals who purchase livestock to eventually take them to slaughter facilities), predominantly in the North region (215; 93.1%) (*p* < 0.05). Furthermore, it is common for participants to also sell to livestock traders (182; 32.4%) and other farmers (154; 27.5%).

Livestock management and biosecurity practices were then assessed to identify possible areas of targeted intervention. Among these small-scale owners, the majority practice semi-intensive farming (664; 56.5%), where animals are occasionally kept within a fenced area but are also allowed to graze and find water freely. Transhumance, or nomadic pastoralism, is also common (273; 23.2%), with animals never kept within a fenced area and traveling long distances for food and water. Extensive farming is also practiced (211; 17.9%), where animals are never fenced but remain on the owner’s property. Within these production systems, 977 (88.2%) of the farmers comingle their animals with those of other owners, and 994 (84.5%) share a water source with other farmers.

Reproductive waste is a major mechanism of *Brucella* transmission. Only 348 (29.6%) of livestock farmers indicated that they handle animal waste after birth, with most (318; 91.6%) washing their hands afterward. However, only 490 (43.1%) of farmers bury aborted material, primarily in the Far North (261; 61.3%) and North (181; 52.5%) over the West (48; 13.1%) (*p* < 0.05). A substantial portion (361; 31.8%) give these materials to dogs, while 338 (29.7%) leave them in place. Only one individual reported using burning as a disposal method. Additionally, when their animals are sick, farmers predominantly seek veterinary services (861; 88.9%) for treatment, rather than self-treating with unregulated drugs from local markets (107; 11.1%) (*p* < 0.05). Furthermore, only 116 (9.9%) indicated they would sell a constantly sick animal to another farmer, or 212 (18.1%) would keep the animal until it recovered without providing healthcare, both practices being more common in the North compared to the Far North and West (*p* < 0.05). Generally, farmers would sell these animals for slaughter (782; 66.7%), keep them until they recover with healthcare, or slaughter them at home (574; 49.0%).

Slaughter can contribute to the transmission of *Brucella*, primarily due to mechanical aerosolization during the process and the handling of infected reproductive tissues. Among the participants, 660 (54.8%) join in the slaughter process of their livestock, primarily in the North (297; 75.8%), followed by the West (216; 57.3%), and the Far North (147; 33.7%) (*p* < 0.05). Only 379 (57.6%) of the farmers wash their hands with soap after slaughter. When slaughtering at home, farmers mainly slaughter goats (717; 59.0%), sheep (556; 45.7%), cattle (257; 21.1%), and pigs (129; 10.6%) (*p* < 0.05). Home slaughter of cattle is predominantly done in the North (197; 50.0%) compared to the West (36; 9.5%) and the Far North (24; 5.4%) (*p* < 0.05).

An evaluation of the farmers’ fundamental knowledge of brucellosis was then conducted to evaluate their awareness and understanding of the disease, its transmission, and the implications of infection. When asked if they had heard of brucellosis or *Baakaale* (the local name for the disease in the Fulani language), only 420 (35.5%) of the farmers responded affirmatively. Of these, 260 (62.2%) expressed concern about the disease, and 366 (87.1%) could identify cattle as susceptible to it. Additionally, 77 (18.3%) identified sheep, 56 (13.3%) identified goats, and 24 (5.7%) identified pigs as animals that can contract the disease. When asked about the symptoms of brucellosis in animals, 214 (51.0%) mentioned hygromas, 169 (40.2%) mentioned abortion, and 127 (30.2%) mentioned being born weak, while 51 (12.1%) admitted they did not know. Furthermore, when asked how animals contract the disease, 266 (63.6%) did not know, with the highest numbers from the Far North (149; 97.9%), followed by the West (108; 78.3%), and the North (9; 7.3%) (*p* < 0.05). Although farmers from the North were more likely to respond to the question, only 99 (80.5%) to 43 (35.0%) people correctly identified various transmission pathways, while many (32; 26.0% to 23; 18.7%) incorrectly identified feces, cough, and blood as transmission methods. Interestingly, 484 (41.2%) of all farmers expressed no concern about any other infectious livestock diseases.

Human brucellosis is common in areas where the disease is endemic in livestock. An understanding of this is important for the public health prevention of disease. When farmers who had heard of brucellosis were asked if humans could contract the disease, 258 (61.4%) did not know, 115 (27.4%) answered “no,” and only 47 (11.2%) responded “yes.” The North had the highest number of farmers aware that humans could get the disease (36; 29.3%), compared to the Far North (6; 3.8%) and the West (5; 3.6%) (*p* < 0.05). Among those who knew about human brucellosis, 32 (91.4%) in the North identified raw milk as a risk, and 30 (85.7%) identified reproductive waste, but 21 (60.0%) incorrectly identified animal feces.

Raw (unboiled) milk is a primary vector for *Brucella* transmission to the general population, so we wanted to further investigate the farmers’ KAP and evaluate their milk-related practices. Although only 258 (21.9%) of these farmers sell or trade milk nationwide, this practice is most common in the North (139; 39.7%), followed by the West (79; 21.0%) and the Far North (40; 8.9%) (*p* < 0.05). Countrywide, only 82 (31.9%) of these farmers boil the milk before selling it. Furthermore, farmers in the North (25; 18.0%) boil their milk at a significantly lower rate compared to those in the West (40; 51.3%) and the Far North (17; 42.5%) (*p* < 0.05). Markets are the primary point of sale (165; 94.3%). Additionally, it is rare for these farmers to sell products made from milk (60; 5.1%), but the majority (56; 93.3%) boil the milk before making these products.

Most livestock owners and their families consume milk (1,059; 86.4%), with 532 (56.8%) drinking milk from their own animals, and 281 (52.9%) boiling it. Among these milk drinkers, 608 (57.5%) consume milk from other people’s livestock, with 427 (70.6%) purchasing it from other farms and 292 (48.3%) buying it from local markets. Additionally, only 375 (35.6%) have access to commercially produced milk, and the majority of them (311; 82.9%) drink this milk. Furthermore, only 71 (5.8%) consume products made from milk, and all 9 individuals who produce these products boil the milk before making them. Interestingly, the majority of farmers who drink milk and know that raw milk can transmit brucellosis to humans still choose not to boil it (16; 51.6%).

### 3.2 Livestock trader survey

Livestock traders act as intermediaries, facilitating trade between farms and abattoirs, and can contribute to the spread of *Brucella* beyond farms and across the country. Surveys of these traders were conducted at slaughter facilities and livestock markets to identify areas of potential targeted intervention, with the findings presented in Supplementary Table 2. Similar to livestock owners, livestock traders are predominantly male (1,132; 98.3%) and mostly fall within the age range of 38 to 57 years (961; 83.8%). A substantial portion of the traders are illiterate (613; 54.8%), and 525 (46.0%) have no formal education. Most participants are small-scale livestock traders, purchasing five or fewer animals at a time, acquiring new livestock weekly (681; 59.6%) to daily (364; 31.8%), from three or fewer live markets (1,086; 95.0%), with most purchases occurring at live markets (1,053; 92.0%). Livestock are mainly purchased for live trade in the West (364; 93.8%), followed by the Far North (291; 75.6%) and the North (8; 2.1%) (*p* < 0.05). In the North, conversely, livestock are predominantly bought for future slaughter (371; 99.2%), followed by the Far North (96; 24.9%) and the West (66; 17.0%) (*p* < 0.05). Among these traders, 573 (50.0%) buy and sell goats, followed by sheep (542; 47.3%), cattle (479; 41.8%), and pigs (125; 10.9%).

An assessment of livestock management and biosecurity practices was carried out to identify probable deficiencies for targeted intervention and to evaluate the potential risk of disease spread. Most often, traders transport animals by walking them to their destination (764; 67.6%). Traders typically sell or slaughter livestock on a daily (476; 42.1%), weekly (380; 33.6%), or monthly (250; 22.1%) basis, usually keeping them for 10 days or less (778; 67.8%). Comingling of livestock occurs throughout the trade process. Prior to purchase, livestock are normally kept in an open pasture (633; 58.9%) or fenced area (329; 29.2%) that belongs to someone else. After acquisition, 561 (49.2%) keep them in a pen that belongs to someone else or 398 (34.9%) in self-owned pens, while 623 (54.8%) keep their livestock along with animals that they do not own, predominantly in the West (283; 72.9%), followed by the Far North (214; 55.6%) and the North (126; 34.6%) (*p* < 0.05).

Basic disinfection practices, such as cleaning footwear and washing hands before entering new facilities or handling animals, were evaluated. A common way diseases spread between locations is through the feet of individuals who enter and exit without sanitizing them. Wearing clean boots is essential for preventing the spread of disease between different locations. During farm, market, and slaughter facility visits, most traders do not wear a specific set of clean shoes (1,047; 92.7%). However, individuals who use a designated set of boots usually wash them with soap and water before both entering and exiting (59; 73.8%). Additionally, most traders do not consistently wash their hands with soap and water before entering and leaving these locations (1,015; 90.2%).

Traders serve as intermediaries for both milk and livestock. To further investigate knowledge of *Brucella* transmission via milk, traders were also asked if drinking raw (unboiled) milk could transmit certain animal diseases to people, and 742 (66.6%) did not know, 353 (30.2%) responded “no,” and only 33 (3.0%) responded “yes.”

### 3.3 Slaughter facility inspector survey

To understand the knowledge, attitudes, and practices (KAP) beyond the supply chain and within the regulatory body, all governmental meat inspectors at the abattoir sample sites were surveyed. This was done to identify areas for education and targeted intervention to mitigate risk. The results are provided in Supplementary Table 3. However, with only 36 inspectors, the statistical power was insufficient to compare proportions between regions. Despite this, a relatively even number of male (19; 52.8%) and female (17; 47.2%) inspectors were represented, with the majority (23; 63.9%) having been inspectors for less than 10 years, while 13 (36.1%) had less than 5 years of experience. These inspectors primarily worked at either low throughput facilities (14; 45.2%), where they would inspect fewer than 10 animals per day, or high throughput facilities (10; 32.3%), where they would inspect 50 or more animals per day. Countrywide, inspectors mainly inspected cattle (24; 66.7%), followed by goats (12; 33.3%), sheep (11; 30.6%), and pigs (7; 19.4%). Regional differences were observed, with inspectors in the Far North predominantly checking goats and sheep, while those in the North and West primarily examined cattle.

To assess whether inspectors could identify brucellosis in livestock during the slaughter process, we asked them about the timing of their inspections. At these facilities, only 18 (50.0%) of the inspectors conducted pre-slaughter disease inspections countrywide, with 18 (50.0%) inspectors in the North and 5 (50.0%) in the Far North. In the North, the majority of inspectors (10; 83.3%) checked the animals, while most inspectors in the West (11; 78.6%) did not. Additionally, most inspectors performed disease inspections during slaughter, with 26 (72.2%) doing so countrywide, 13 (92.9%) in the West, 8 (66.7%) in the North, and 5 (50.0%) in the Far North.

Given that *B. abortus* is the only endemic *Brucella* species in Cameroon (12, 13), this analysis primarily focuses on assessing inspector knowledge of the disease in cattle. Results for goats, sheep, and pigs are provided in Supplementary Table 3. To determine if inspectors could recognize brucellosis, we assessed their knowledge of the disease’s signs. Unlike livestock owners and traders, almost all slaughter facility meat inspectors reported having heard of brucellosis, with 34 (94.4%) countrywide, 12 (100%) in the North, 13 (92.9%) in the West, and 9 (90.0%) in the Far North. Despite this awareness, only 29 (82.9%) countrywide could correctly identify abortion as a possible sign of the disease in live cattle, while 24 (68.6%) identified hygromas, and 22 (62.9%) identified reduced fertility. Additionally, only 2 (5.7%) reported not knowing any possible signs in cattle, but common incorrect answers included bloody manure/feces, difficult breathing, and red eyes with tears. Furthermore, although most inspectors examined animals during the slaughter process, only 13 (38.2%) countrywide identified enlarged lymph nodes, 10 (30.3%) identified lesions in the placenta, and 7 (21.2%) identified lesions in the uterus. In the Far North, 5 (50.0%) reported not knowing any possible post-mortem signs, and the other 5 (50.0%) said there are no signs. Common misconceptions countrywide included swollen or dark mammary glands/udders and widespread bleeding over organ and body surfaces. When asked about differential diagnoses, 15 (45.5%) countrywide reported not knowing, and 7 (21.2%) said there is no differential diagnosis. Other misconceptions included contagious bovine pleuropneumonia (CBPP) and foot-and-mouth disease (FMD). Although inspector knowledge of brucellosis in goats, sheep, and pigs is not directly discussed here, evidence in Supplementary Table 3 indicates an equivalent to lesser understanding than that for cattle.

Inspectors are not only responsible for identifying diseases in livestock, but also for determining the fate of the animals or products, so their management and biosecurity practices were then assessed. When asked about their usual practices, the majority (20; 55.6%) declined to answer how often they identify possible cases of brucellosis in live animals countrywide, while 8 (22.2%) indicated an annual rate. If they suspect a live animal has brucellosis, 16 (44.4%) would allow it to be slaughtered, 11 (30.6%) would reject it, and 9 (25.0%) declined to respond. Regarding the identification of brucellosis in slaughtered animals, 25 (69.4%) declined to answer, while 6 (16.7%) indicated an annual rate. If they suspect a slaughtered animal has brucellosis, 20 (55.6%) would condemn the part with lesions, 6 (16.7%) would allow the entire animal to be processed, and 4 (11.1%) would condemn the entire animal. When a slaughtered animal is condemned due to disease, 23 (63.9%) would bury the carcass, while 21 (58.3%) would burn it. Additionally, when fetuses and placentas of pregnant animals are identified during slaughter, 15 (46.9%) would give them to dogs or wild animals, 15 (46.9%) would bury them, and 7 (21.9%) would burn them. The North was the only region where inspectors did not report giving them to dogs or wild animals. Furthermore, when asked if they would contact LANAVET (the institution responsible for diagnostic testing and surveillance) if they suspect an animal has brucellosis, only 13 (36.1%) indicated that they would.

Handling infected tissues greatly contributes to the transmission of *Brucella.* Diseases commonly spread between locations through the hands of individuals who do not sanitize them. Using washable or disposable gloves on both hands and thoroughly washing hands between locations can reduce the risk of disease transmission. These meat inspectors handle livestock and carcasses continually throughout the day, but when asked about their usual occupational hygiene practices, only 20 (55.6%) reported always wearing rubber gloves on both hands, and 12 (75.0%) always dispose of the gloves after use. Additionally, 29 (80.6%) always wash their hands after work, and 28 (77.8%) always remove and wash their clothes/aprons. Furthermore, 29 (80.6%) reported always wearing rubber boots while working, and 26 (89.7%) of those individuals always wash their boots after work.

## 4 Discussion

*Brucella* spp. has long been recognized as endemic across Africa, making it the highest-risk region for human brucellosis globally (4). However, the full extent and impact of the disease remain poorly understood, especially in West Africa (16). Recent studies have shown that *B. abortus* is the endemic *Brucella* species in Cameroon, found in livestock (mainly cattle) at slaughter facilities and in milk sold at open community markets across the country (12, 13). While public knowledge and perception of diseases play a critical role in prevention, particularly in endemic areas, developing and implementing effective control and management strategies requires a comprehensive understanding of the local population’s KAP regarding the disease. Therefore, recognizing brucellosis as an occupational disease and the importance of biosecurity practices for its control and prevention, this study establishes a baseline of animal workers’ KAP regarding brucellosis. By assessing their understanding of the disease, their attitudes toward potentially infected animals, and their occupational hygiene practices, the study seeks to identify areas needing targeted interventions. Specifically, this study presents findings from three KAP surveys conducted among (1) livestock owners, (2) livestock traders, and (3) slaughter facility inspectors. Although previous research in the country has focused on addressing knowledge gaps regarding livestock prevalence and public health risks linked to the open sale of contaminated milk (12, 13), no studies, to our knowledge, have assessed the understanding of brucellosis among these three specific populations at locations where *Brucella* has been isolated and characterized.

In Cameroon, as in many African countries, meat is primarily sourced from local markets, with small-scale farms playing a significant role in the supply chain. Meat for home consumption is typically obtained from two main sources: (1) local open-air community slaughter facilities and markets, where small-scale farmers and livestock traders sell animal products directly to the public (the primary source), and (2) supermarkets, which offer commercially produced products. Consequently, local slaughter facilities and markets where *Brucella* has been isolated and characterized were selected as survey sites. To evaluate the KAP within farmer and trader populations, a random selection of these animal workers was given a structured, close-ended survey at these sites. The surveys were analyzed to assess their basic knowledge of the disease, as well as specific occupational and biosecurity risk behaviors relevant to their roles. Additionally, all governmental meat inspectors were surveyed, and the data was analyzed to assess their fundamental understanding of disease signs and pertinent occupational and biosecurity risk behaviors related to their roles.

### 4.1 Livestock owners

The survey conducted among livestock owners at slaughter facilities and livestock markets provides a comprehensive overview of the demographics, livestock management practices, and biosecurity measures in place. The findings highlight several key areas that could benefit from targeted interventions to improve livestock and human health and reduce the transmission of brucellosis.

The evaluation of farmers’ knowledge about brucellosis reveals a significant lack of awareness and understanding regarding the disease, its transmission, and its implications. Many farmers are unaware that humans can contract brucellosis, and there is confusion about how the disease spreads. This highlights the urgent need for educational campaigns to raise awareness and improve knowledge about brucellosis among livestock owners. The assessment identifies several areas of concern in farmers’ biosecurity practices, including inadequate handling of reproductive waste, reliance on unregulated drugs, insufficient handwashing, and the failure to boil milk before sale. Addressing these practices could significantly reduce the risk of *Brucella* transmission within and between farms, as well as to the general population.

Livestock ownership in Cameroon is predominantly held by middle-aged males, most of whom have limited formal education. This demographic profile suggests that educational programs tailored to this group could be particularly effective. Additionally, a significant portion of livestock is sold for purposes other than slaughter, with butchers being the primary buyers, indicating a well-established market for live animals, which could be leveraged to promote better health and biosecurity practices.

### 4.2 Livestock traders

The survey of livestock traders at slaughter facilities and livestock markets provides valuable insights into their role in the livestock trade and the potential for *Brucella* transmission. Similar to livestock owners, traders are predominantly middle-aged males, with many lacking formal education. This demographic suggests that targeted educational programs could be beneficial in improving biosecurity practices among traders.

Livestock traders in Cameroon primarily operate on a small scale, purchasing a few animals at a time from live markets. The frequent acquisition of new livestock, often on a weekly or daily basis, highlights the dynamic nature of the livestock trade. Regional differences in the purpose of livestock purchases (live trade in the West and Far North versus future slaughter in the North) indicate varying market demands and practices that could influence disease transmission dynamics.

The assessment of biosecurity practices among traders reveals several areas of concern, including the frequent comingling of livestock throughout the trade process, inadequate use of clean footwear, and inconsistent handwashing practices. These findings underscore the need for interventions to improve hygiene practices among traders. Additionally, similar to farmers, the survey results indicate a significant gap in traders’ knowledge about *Brucella* transmission via raw milk. The majority of traders are unaware that drinking raw milk can transmit animal diseases to humans. This lack of awareness highlights the need for educational campaigns to inform traders (and likely the general public) about the risks associated with raw milk and the importance of boiling milk before consumption.

### 4.3 Slaughter facility inspectors

The survey of governmental meat inspectors at abattoir sample sites aimed to understand their KAP regarding brucellosis. This effort was also crucial for identifying areas needing education and targeted interventions to mitigate risks and provided valuable insights into the demographics and experience levels of the inspectors.

Most inspectors had less than 10 years of experience, indicating a relatively young workforce. They primarily worked at either low throughput facilities, inspecting fewer than 10 animals per day, or high throughput facilities, inspecting 50 or more animals per day which highlights the varied operational scales between the abattoirs. Inspectors’ practices varied significantly across regions. Only half of the inspectors conducted pre-slaughter disease inspections, with notable regional differences. For instance, inspectors in the North were more likely to perform pre-slaughter checks compared to those in the West. During slaughter, a higher proportion of inspectors conducted disease inspections, particularly in the West. This variation underscores the need for standardized inspection protocols to ensure consistent disease detection.

While most inspectors were aware of brucellosis, their ability to identify its signs varied. A majority could recognize abortion as a sign, but fewer identified other symptoms like hygromas and reduced fertility. Misconceptions about brucellosis signs were common, indicating gaps in knowledge that could affect disease management. Inspectors’ ability to identify brucellosis during post-mortem inspections was limited. Few could recognize enlarged lymph nodes or lesions in the placenta and uterus, with significant regional disparities. This suggests a need for enhanced training on post-mortem signs of brucellosis to improve disease detection and management.

The survey also assessed inspectors’ management and biosecurity practices. Responses differed widely, with many inspectors declining to answer questions about their practices. When brucellosis was suspected in slaughtered animals, inspectors’ disposal methods for potentially contaminated tissues contrasted, with some practices potentially contributing to disease transmission. Occupational hygiene practices among inspectors also conflicted, with most reporting regular use of gloves, hand washing, and cleaning of work attire and boots; however, there is substantial room for improvement, particularly in ensuring consistent use of disposable gloves and proper sanitation between locations.

### 4.4 Risk factors and regional insights

To further explore potential predictors of knowledge, attitudes, and practices related to brucellosis, we conducted a multivariate analysis using survey responses from livestock owners, traders, and slaughterhouse inspectors. The goal was to identify whether any known risk factors—such as education level, occupation, region, or livestock type—were significantly associated with biosecurity practices or disease awareness. However, the analysis did not reveal any strong or moderate associations between these variables. This suggests that the risk factors assessed may operate independently or that other unmeasured variables, such as cultural beliefs or informal knowledge networks, may play a more influential role. These findings underscore the complexity of behavioral determinants in zoonotic disease prevention and highlight the need for broader, context-specific approaches to intervention.

The findings from Cameroon are consistent with high-impact studies conducted across sub-Saharan Africa, which collectively highlight widespread gaps in knowledge and risky practices among livestock handlers (16–21). In Ethiopia, a study in the West Hararghe Zone revealed that 80% of cattle owners had no prior knowledge of brucellosis, 73% engaged in risk behaviors such as handling reproductive waste without protection, and 83% held negative attitudes toward disease prevention (19). In Kenya, despite over 70% of pastoralist and mixed-farming households having heard of brucellosis, fewer than 30% understood its transmission or prevention (21). Notably, nomadic pastoralists were significantly more likely to engage in high-risk behaviors, including consuming raw milk and assisting in animal births without protection (21). In Nigeria, brucellosis remains a neglected zoonosis, with poor public health infrastructure and limited awareness among livestock handlers contributing to its persistence (16). A recent review emphasized the occupational risks faced by farmers and abattoir workers, particularly due to inadequate protective measures and lack of disease education (18). These parallels underscore the urgent need for regionally coordinated interventions that combine public education, occupational hygiene training, and improved veterinary infrastructure to mitigate the zoonotic and economic burden of brucellosis across Africa.

## 5 Conclusion

This comprehensive survey of livestock owners, traders, and governmental meat inspectors at slaughter facilities and livestock markets provides critical insights into the demographics, livestock management practices, and biosecurity measures currently in place. The findings highlight several key areas that require targeted interventions to improve livestock and human health and reduce the transmission of brucellosis. There is a clear need for comprehensive educational and training programs tailored to the specific demographics and practices of livestock owners, traders, and meat inspectors. By addressing the identified gaps in knowledge and biosecurity practices, these interventions can enhance the overall health and productivity of livestock populations, reduce the transmission of brucellosis, and ultimately improve public health outcomes. Collaboration between veterinary and public health professionals is essential for delivering information at the community level to reduce the disease’s prevalence. The evidence gathered in this study is expected to provide clearer insights into the animal and public health risks from farm to slaughter, ultimately aiding policymakers in developing new educational initiatives and interventions to reduce the disease burden.

## Data Availability

The raw data supporting the conclusions of this article will be made available by the authors, without undue reservation.
