# Supplementary material for: Knowledge, attitudes, and practices among livestock owners, traders, and slaughterhouse inspectors in Cameroon reveals marginal understanding of livestock and human brucellosis
Source: Front Vet Sci. 2026 Jan 30;12:1677520. doi: 10.3389/fvets.2025.1677520 (PMC12900696; doi:10.3389/fvets.2025.1677520)
Supplement: Supplementary file 1 [file Table_1.DOCX]

**Supplementary Table 1: Cameroonian livestock owner survey questions and results**

|  | | **Countrywide** | | **Far North** | | **North** | | **West** | |
| --- | --- | --- | --- | --- | --- | --- | --- | --- | --- |
| **Survey Question** | **Answer** | **Frequency │ Rate (%)** | | **Frequency │ Rate (%)** | | **Frequency │ Rate (%)** | | **Frequency │ Rate (%)** | |
| **Number of Livestock Owners** | Livestock Owners | 1,230 | 100% | 455 | 100% | 396 | 100% | 379 | 100% |
| **1. Gender (Choose One)** | Male | 1,181 | 96.0% | 444 | 97.6% | 359 | 90.7% | 378 | 99.7% |
|  | Female | 49 | 4.0% | 11 | 2.4% | 37 | 9.3% | 1 | 0.3% |
| **2. What is your marital status? (Choose One)** | Married | 1,039 | 88.4% | 423 | 95.3% | 250 | 70.4% | 366 | 97.1% |
|  | Single | 137 | 11.6% | 21 | 4.7% | 105 | 29.6% | 11 | 2.9% |
| **3. What is your approximate age? (Enter a Number)** | 18-27 | 7 | 0.6% | 3 | 0.7% | 3 | 0.8% | 1 | 0.3% |
|  | 28-37 | 141 | 11.5% | 85 | 18.7% | 40 | 10.1% | 16 | 4.2% |
|  | 38-47 | 621 | 50.5% | 297 | 65.3% | 134 | 33.8% | 190 | 50.1% |
|  | 48-57 | 369 | 30.0% | 49 | 10.8% | 176 | 44.4% | 144 | 38.0% |
|  | 58-67 | 83 | 6.7% | 16 | 3.5% | 42 | 10.6% | 25 | 6.6% |
|  | 68+ | 9 | 0.7% | 5 | 1.1% | 1 | 0.3% | 3 | 0.8% |
| **4. Which ethnic group do you identify with? (Enter a Group)** | Peul | 357 | 29.0% | 109 | 24.0% | 112 | 28.3% | 136 | 35.9% |
|  | Bamum | 137 | 11.1% | 2 | 0.4% | 0 | 0.0% | 135 | 35.6% |
|  | Tupuri | 96 | 7.8% | 50 | 11.0% | 46 | 11.6% | 0 | 0.0% |
|  | Masa | 77 | 6.3% | 56 | 12.3% | 20 | 5.1% | 1 | 0.3% |
|  | Fali | 67 | 5.4% | 19 | 4.2% | 46 | 11.6% | 2 | 0.5% |
|  | Laka | 61 | 5.0% | 1 | 0.2% | 60 | 15.2% | 0 | 0.0% |
|  | Guiziga | 47 | 3.8% | 32 | 7.0% | 15 | 3.8% | 0 | 0.0% |
|  | Mbum | 40 | 3.3% | 3 | 0.7% | 37 | 9.3% | 0 | 0.0% |
|  | Mofu | 35 | 2.8% | 35 | 7.7% | 0 | 0.0% | 0 | 0.0% |
|  | Musulman | 35 | 2.8% | 0 | 0.0% | 0 | 0.0% | 35 | 9.2% |
|  | Bamiléké | 30 | 2.4% | 0 | 0.0% | 22 | 5.6% | 8 | 2.1% |
|  | Musgum | 30 | 2.4% | 30 | 6.6% | 0 | 0.0% | 0 | 0.0% |
|  | Hausa | 21 | 1.7% | 0 | 0.0% | 0 | 0.0% | 21 | 5.5% |
|  | Mandara | 20 | 1.6% | 20 | 4.4% | 0 | 0.0% | 0 | 0.0% |
|  | Other (40 different groups) | 177 | 14.4% | 98 | 21.5% | 38 | 9.6% | 41 | 10.8% |
| **5. Currently, what additional sources of household income do you have? (Choose All That Apply)** | Crop Farming | 684 | 57.1% | 284 | 65.6% | 192 | 49.2% | 208 | 72.5% |
|  | Dairy Farmer | 231 | 19.3% | 33 | 7.6% | 102 | 26.2% | 8 | 2.8% |
|  | None | 143 | 11.9% | 97 | 22.4% | 0 | 0.0% | 46 | 16.0% |
|  | Butcher | 116 | 9.7% | 18 | 4.2% | 86 | 22.1% | 12 | 4.2% |
|  | Agent of Butcher | 18 | 1.5% | 0 | 0.0% | 10 | 2.6% | 8 | 2.8% |
|  | Other | 6 | 0.5% | 1 | 0.2% | 0 | 0.0% | 5 | 1.7% |
| **6. Do you know how to read? (Choose One)** | No | 687 | 56.6% | 250 | 54.9% | 194 | 51.2% | 243 | 64.1% |
|  | Yes | 526 | 43.4% | 205 | 45.1% | 185 | 48.8% | 136 | 35.9% |
| **7. What is your highest level of education? (Choose One)** | No formal education | 582 | 47.5% | 168 | 36.9% | 221 | 56.2% | 193 | 51.1% |
|  | Primary school | 231 | 18.8% | 114 | 25.1% | 38 | 9.7% | 79 | 20.9% |
|  | Secondary school | 315 | 25.7% | 119 | 26.2% | 114 | 29.0% | 82 | 21.7% |
|  | Technical School | 67 | 5.5% | 44 | 9.7% | 2 | 0.5% | 21 | 5.6% |
|  | University | 31 | 2.5% | 10 | 2.2% | 18 | 4.6% | 3 | 0.8% |
| **8. Which animals do you own? (Choose All That Apply)** | Goats | 825 | 70.0% | 343 | 76.1% | 265 | 75.7% | 217 | 57.6% |
|  | Sheep | 755 | 64.1% | 290 | 64.3% | 239 | 68.3% | 226 | 59.9% |
|  | Cattle | 643 | 54.6% | 226 | 50.1% | 202 | 57.7% | 215 | 57.0% |
|  | Pigs | 176 | 14.9% | 74 | 16.4% | 72 | 20.6% | 30 | 8.0% |
| **9. How many family members and employees care for your animals? (Choose One)** | 0 | 82 | 7.0% | 0 | 0.0% | 82 | 23.4% | 0 | 0.0% |
|  | 1-5 | 1,061 | 90.1% | 433 | 96.0% | 268 | 76.6% | 360 | 95.5% |
|  | 6-10 | 35 | 3.0% | 18 | 4.0% | 0 | 0.0% | 17 | 4.5% |
|  | 11-15 | 0 | 0.0% | 0 | 0.0% | 0 | 0.0% | 0 | 0.0% |
|  | 16+ | 0 | 0.0% | 0 | 0.0% | 0 | 0.0% | 0 | 0.0% |
| **10. How would you describe the movements of your animals? (Choose One)** | Animals are sometimes kept within a fenced area, but let out to graze and to find water (Semi-Intensive) | 664 | 56.5% | 222 | 49.3% | 159 | 45.4% | 283 | 75.3% |
|  | Animals are never kept within a fenced area, and travel long distance for food and water (Transhumance) | 273 | 23.2% | 121 | 26.9% | 80 | 22.9% | 72 | 19.1% |
|  | Animals are never kept within a fenced area, but stay on your property (Extensive) | 211 | 17.9% | 105 | 23.3% | 95 | 27.1% | 11 | 2.9% |
|  | Animals are always kept within a fenced area (Intensive) | 28 | 2.4% | 2 | 0.4% | 16 | 4.6% | 10 | 2.7% |
| **11. Do your animals come into contact with any of these? (Choose All That Apply)** | Other people’s animals | 977 | 88.2% | 320 | 72.7% | 298 | 99.3% | 359 | 97.6% |
|  | Other animal species that you own | 133 | 12.0% | 121 | 27.5% | 3 | 1.0% | 9 | 2.4% |
|  | Wildlife | 26 | 2.3% | 1 | 0.2% | 25 | 8.3% | 0 | 0.0% |
| **12. Do you share a water source with other farmers? (Choose One)** | Yes | 994 | 84.5% | 373 | 82.7% | 279 | 80.2% | 342 | 90.7% |
|  | No | 182 | 15.5% | 78 | 17.3% | 69 | 19.8% | 35 | 9.3% |
| **13. Generally, how often do you acquire new livestock, and bring them into your heard? (Choose One)** | Never | 21 | 1.8% | 0 | 0.0% | 1 | 0.3% | 20 | 5.3% |
|  | Daily | 2 | 0.2% | 0 | 0.0% | 1 | 0.3% | 1 | 0.3% |
|  | Weekly | 65 | 5.5% | 36 | 8.0% | 0 | 0.0% | 29 | 7.7% |
|  | Monthly | 402 | 34.2% | 188 | 41.8% | 12 | 3.4% | 202 | 53.9% |
|  | Yearly | 170 | 14.5% | 94 | 20.9% | 70 | 20.1% | 6 | 1.6% |
|  | As Needed | 514 | 43.8% | 132 | 29.3% | 265 | 75.9% | 117 | 31.2% |
| **14. Do you share, lend, or borrow animals with other farmers? (Choose One)** | No | 1,049 | 89.4% | 419 | 92.9% | 276 | 79.5% | 354 | 94.1% |
|  | Yes | 125 | 10.6% | 32 | 7.1% | 71 | 20.5% | 22 | 5.9% |
| **14.1. Which of these animals do you share, lend, or borrow? (Choose All That Apply)** | Cattle Male | 65 | 52.0% | 15 | 46.9% | 31 | 43.7% | 19 | 86.4% |
|  | Sheep Male | 58 | 46.4% | 5 | 15.6% | 43 | 60.6% | 10 | 45.5% |
|  | Cattle Female | 20 | 16.0% | 14 | 43.8% | 6 | 8.5% | 0 | 0.0% |
|  | Pigs Male | 19 | 15.2% | 2 | 6.3% | 17 | 23.9% | 0 | 0.0% |
|  | Goats Male | 13 | 10.4% | 1 | 3.1% | 9 | 12.7% | 3 | 13.6% |
|  | Sheep Female | 9 | 7.2% | 9 | 28.1% | 0 | 0.0% | 0 | 0.0% |
|  | Goats Female | 3 | 2.4% | 3 | 9.4% | 0 | 0.0% | 0 | 0.0% |
|  | Pigs Female | 2 | 1.6% | 1 | 3.1% | 1 | 1.4% | 0 | 0.0% |
| **15. Do you ever sell live animals? (Not for slaughter) (Choose One)** | No | 612 | 52.2% | 296 | 65.6% | 114 | 33.0% | 202 | 53.6% |
|  | Yes | 561 | 47.8% | 155 | 34.4% | 231 | 67.0% | 175 | 46.4% |
| **15.1 Do you sell any of these animals live? (Not for slaughter) (Choose All That Apply)** | Cattle | 384 | 68.6% | 112 | 72.7% | 141 | 61.3% | 131 | 74.4% |
|  | Sheep | 354 | 63.2% | 93 | 60.4% | 155 | 67.4% | 106 | 60.2% |
|  | Goats | 320 | 57.1% | 78 | 50.6% | 167 | 72.6% | 75 | 42.6% |
|  | Pigs | 62 | 11.1% | 3 | 1.9% | 48 | 20.9% | 11 | 6.3% |
| **15.2. To whom do you sell these animals? (Enter whom)** | Butchers | 326 | 58.1% | 71 | 45.8% | 215 | 93.1% | 40 | 22.9% |
|  | Livestock traders | 182 | 32.4% | 63 | 40.6% | 17 | 7.4% | 102 | 58.3% |
|  | Farmers | 154 | 27.5% | 87 | 56.1% | 0 | 0.0% | 67 | 38.3% |
|  | Breeders | 77 | 13.7% | 0 | 0.0% | 76 | 32.9% | 1 | 0.6% |
|  | Market | 40 | 7.1% | 0 | 0.0% | 8 | 3.5% | 32 | 18.3% |
| **16. Do you handle animal waste after they give birth? (Choose One)** | No | 827 | 70.4% | 289 | 64.1% | 300 | 86.5% | 238 | 63.1% |
|  | Yes | 348 | 29.6% | 162 | 35.9% | 47 | 13.5% | 139 | 36.9% |
| **16.1. Do you wash your hands after? (Choose One)** | Yes | 318 | 91.6% | 155 | 95.7% | 39 | 84.8% | 124 | 89.2% |
|  | No | 29 | 8.4% | 7 | 4.3% | 7 | 15.2% | 15 | 10.8% |
| **16.2. What do you do with the dead newborn animals? (Choose All That Apply)** | Bury | 490 | 43.1% | 261 | 61.3% | 181 | 52.5% | 48 | 13.1% |
|  | Give to Dogs | 361 | 31.8% | 131 | 30.8% | 130 | 37.7% | 100 | 27.3% |
|  | Leave it in place | 338 | 29.7% | 39 | 9.2% | 73 | 21.2% | 226 | 61.7% |
|  | Burn | 1 | 0.1% | 0 | 0.0% | 1 | 0.3% | 0 | 0.0% |
| **17. How do you treat sick animals? (Choose One)** | Veterinary Services | 861 | 88.9% | 380 | 84.3% | 139 | 99.3% | 342 | 90.7% |
|  | Self treatment (Drugs from market) | 107 | 11.1% | 71 | 15.7% | 1 | 0.7% | 35 | 9.3% |
| **18. What do you do with animals that are constantly sick? (Choose All That Apply)** | Sell for slaughter | 782 | 66.7% | 366 | 81.3% | 165 | 48.0% | 251 | 66.4% |
|  | Keep until they are better (With Healthcare) | 593 | 50.6% | 293 | 65.1% | 89 | 25.9% | 211 | 55.8% |
|  | Home slaughter | 574 | 49.0% | 225 | 50.0% | 195 | 56.7% | 154 | 40.7% |
|  | Keep until they are better (Without Healthcare) | 212 | 18.1% | 45 | 10.0% | 137 | 39.8% | 30 | 7.9% |
|  | Sell to another farmer | 116 | 9.9% | 39 | 8.7% | 59 | 17.2% | 18 | 4.8% |
| **19. Do you ever sell/trade milk? (Choose One)** | No | 920 | 78.1% | 411 | 91.1% | 211 | 60.3% | 298 | 79.0% |
|  | Yes | 258 | 21.9% | 40 | 8.9% | 139 | 39.7% | 79 | 21.0% |
| **19.1. Do you boil the milk before you sell it? (Choose One)** | No | 175 | 68.1% | 23 | 57.5% | 114 | 82.0% | 38 | 48.7% |
|  | Yes | 82 | 31.9% | 17 | 42.5% | 25 | 18.0% | 40 | 51.3% |
| **19.2. Who do you sell the milk to? (Enter Whom)** | Market | 165 | 94.3% | 17 | 73.9% | 112 | 98.2% | 36 | 94.7% |
|  | Industry | 7 | 4.0% | 5 | 21.7% | 0 | 0.0% | 2 | 5.3% |
|  | Other Farmers | 3 | 1.7% | 1 | 4.3% | 2 | 1.8% | 0 | 0.0% |
| **20. Do you ever sell products made from milk? (Choose One)** | No | 1,115 | 94.9% | 427 | 94.7% | 337 | 97.1% | 351 | 93.1% |
|  | Yes | 60 | 5.1% | 24 | 5.3% | 10 | 2.9% | 26 | 6.9% |
| **20.1. Do you boil the milk before making these products? (Choose One)** | Yes | 56 | 93.3% | 24 | 100% | 6 | 60.0% | 26 | 100% |
|  | No | 4 | 6.7% | 0 | 0.0% | 4 | 40.0% | 0 | 0.0% |
| **20.2. Who do you sell these products to? (Enter Whom)** | Market | 4 | 100% | 0 | 0.0% | 4 | 100% | 0 | 0.0% |
| **21. Do you ever slaughter any of these animals at home? (Choose All That Apply)** | Goats | 717 | 59.0% | 263 | 59.1% | 276 | 70.1% | 178 | 47.2% |
|  | Sheep | 556 | 45.7% | 141 | 31.7% | 250 | 63.5% | 165 | 43.8% |
|  | Cattle | 257 | 21.1% | 24 | 5.4% | 197 | 50.0% | 36 | 9.5% |
|  | None | 249 | 20.5% | 135 | 30.3% | 7 | 1.8% | 107 | 28.4% |
|  | Pigs | 129 | 10.6% | 24 | 5.4% | 93 | 23.6% | 12 | 3.2% |
| **22. Do you ever participate in the slaughter process? (Choose One)** | Yes | 660 | 54.8% | 147 | 33.7% | 297 | 75.8% | 216 | 57.3% |
|  | No | 545 | 45.2% | 289 | 66.3% | 95 | 24.2% | 161 | 42.7% |
| **22.1. Do you ever: (Choose All That Apply)** | Wash your hands with soap after slaughter | 379 | 57.6% | 87 | 60.0% | 192 | 64.6% | 100 | 46.3% |
|  | Neither of these | 279 | 42.4% | 58 | 40.0% | 105 | 35.4% | 116 | 53.7% |
|  | Wear a mask during slaughter | 3 | 0.5% | 0 | 0.0% | 3 | 1.0% | 0 | 0.0% |
| **23. Have you heard of brucellosis (baakaale)? (Choose One)** | No | 763 | 64.5% | 295 | 65.1% | 228 | 65.0% | 240 | 63.3% |
|  | Yes | 420 | 35.5% | 158 | 34.9% | 123 | 35.0% | 139 | 36.7% |
| **23.1. Are you concerned about brucellosis (baakaale)? (Choose One)** | Yes | 260 | 62.2% | 98 | 62.0% | 105 | 86.8% | 57 | 41.0% |
|  | No | 158 | 37.8% | 60 | 38.0% | 16 | 13.2% | 82 | 59.0% |
| **23.2. Which animals get this disease? (Some are correct and some are incorrect) (Choose All That Apply)** | Cattle | 366 | 87.1% | 127 | 80.4% | 113 | 91.9% | 126 | 90.6% |
|  | Sheep | 77 | 18.3% | 14 | 8.9% | 57 | 46.3% | 6 | 4.3% |
|  | Goats | 56 | 13.3% | 11 | 7.0% | 41 | 33.3% | 4 | 2.9% |
|  | I do not know | 43 | 10.2% | 24 | 15.2% | 6 | 4.9% | 13 | 9.4% |
|  | Pigs | 24 | 5.7% | 0 | 0.0% | 24 | 19.5% | 0 | 0.0% |
|  | Dogs | 10 | 2.4% | 0 | 0.0% | 10 | 8.1% | 0 | 0.0% |
|  | Bats | 2 | 0.5% | 0 | 0.0% | 2 | 1.6% | 0 | 0.0% |
| **23.3. Which are the symptoms of the disease in animals? (Some are correct and some are incorrect) (Choose All That Apply)** | Swollen Leg Joint | 214 | 51.0% | 49 | 31.0% | 100 | 81.3% | 65 | 46.8% |
|  | Born Dead | 169 | 40.2% | 88 | 55.7% | 39 | 31.7% | 42 | 30.2% |
|  | Born Weak | 127 | 30.2% | 33 | 20.9% | 56 | 45.5% | 38 | 27.3% |
|  | I do not know | 51 | 12.1% | 18 | 4.3% | 5 | 1.2% | 28 | 6.7% |
|  | Won’t get pregnant | 49 | 11.7% | 23 | 14.6% | 26 | 21.1% | 0 | 0.0% |
|  | Red/blotchy skin lesions | 44 | 10.5% | 4 | 2.5% | 40 | 32.5% | 0 | 0.0% |
|  | Swollen testicles | 37 | 8.8% | 0 | 0.0% | 35 | 28.5% | 2 | 1.4% |
|  | Blood in feces | 22 | 5.2% | 0 | 0.0% | 22 | 17.9% | 0 | 0.0% |
|  | Difficulty breathing | 15 | 3.6% | 1 | 0.6% | 14 | 11.4% | 0 | 0.0% |
|  | Swollen Eyes | 10 | 2.4% | 0 | 0.0% | 10 | 8.1% | 0 | 0.0% |
| **23.4. How do animals get the disease? (Some are correct and some are incorrect) (Choose All That Apply)** | I do not know | 266 | 63.6% | 149 | 94.9% | 9 | 7.3% | 108 | 78.3% |
|  | Contact with other animals | 127 | 30.4% | 2 | 1.3% | 99 | 80.5% | 26 | 18.8% |
|  | Stuff leftover after dead birth | 74 | 17.7% | 1 | 0.6% | 69 | 56.1% | 4 | 2.9% |
|  | Milk | 66 | 15.8% | 0 | 0.0% | 66 | 53.7% | 0 | 0.0% |
|  | Urine | 65 | 15.6% | 0 | 0.0% | 65 | 52.8% | 0 | 0.0% |
|  | Reproduction | 53 | 12.7% | 6 | 3.8% | 43 | 35.0% | 4 | 2.9% |
|  | Feces | 32 | 7.7% | 0 | 0.0% | 32 | 26.0% | 0 | 0.0% |
|  | Cough | 24 | 5.7% | 0 | 0.0% | 24 | 19.5% | 0 | 0.0% |
|  | Blood | 23 | 5.5% | 0 | 0.0% | 23 | 18.7% | 0 | 0.0% |
| **23.5 Can humans get the disease? (Choose One)** | I do not know | 258 | 61.4% | 114 | 72.2% | 66 | 53.7% | 78 | 56.1% |
|  | No | 115 | 27.4% | 38 | 24.1% | 21 | 17.1% | 56 | 40.3% |
|  | Yes | 47 | 11.2% | 6 | 3.8% | 36 | 29.3% | 5 | 3.6% |
| **23.5.1. How do humans get the disease? (Some are correct and some are incorrect) (Choose All That Apply)** | Raw Milk (Not Boiled) | 39 | 84.8% | 6 | 100% | 32 | 91.4% | 1 | 20.0% |
|  | Contact with stuff left over after dead birth | 31 | 67.4% | 0 | 0.0% | 30 | 85.7% | 1 | 20.0% |
|  | Animal Feces | 21 | 45.7% | 0 | 0.0% | 21 | 60.0% | 0 | 0.0% |
|  | I do not know | 5 | 10.9% | 0 | 0.0% | 2 | 5.7% | 3 | 60.0% |
|  | Water | 4 | 8.7% | 2 | 33.3% | 2 | 5.7% | 0 | 0.0% |
|  | Meat | 1 | 2.2% | 0 | 0.0% | 1 | 2.9% | 0 | 0.0% |
|  | Mosquitoes | 1 | 2.2% | 0 | 0.0% | 1 | 2.9% | 0 | 0.0% |
|  | Other People | 0 | 0.0% | 0 | 0.0% | 0 | 0.0% | 0 | 0.0% |
| **24. Which animal diseases are you concerned with? (Choose All That Apply)** | I am not concerned | 484 | 41.2% | 172 | 37.9% | 150 | 43.9% | 162 | 42.9% |
|  | Parasites | 379 | 32.3% | 135 | 29.7% | 112 | 32.7% | 132 | 34.9% |
|  | Contagious bovine pleuropneumonia (CBPP) | 285 | 24.3% | 137 | 30.2% | 92 | 26.9% | 56 | 14.8% |
|  | Foot-and-mouth disease (FMD) | 237 | 20.2% | 108 | 23.8% | 57 | 16.7% | 72 | 19.0% |
|  | Bovine Tuberculosis | 176 | 15.0% | 67 | 14.8% | 84 | 24.6% | 25 | 6.6% |
|  | Peste des petits ruminants (PPR) | 82 | 7.0% | 9 | 2.0% | 70 | 20.5% | 3 | 0.8% |
|  | African swine fever (ASF) | 66 | 5.6% | 5 | 1.1% | 47 | 13.7% | 14 | 3.7% |
|  | Contagious caprine pleuropneumonia (CCPP) | 36 | 3.1% | 4 | 0.9% | 31 | 9.1% | 1 | 0.3% |
| **25. Do you or your family drink milk? (Choose One)** | Yes | 1,059 | 86.4% | 384 | 84.4% | 373 | 95.4% | 302 | 79.7% |
|  | No | 166 | 13.6% | 71 | 15.6% | 18 | 4.6% | 77 | 20.3% |
| **25.1. Do you or your family ever drink milk from your own animals? (Choose One)** | Yes | 532 | 56.8% | 156 | 50.6% | 182 | 49.5% | 194 | 74.6% |
|  | No | 404 | 43.2% | 152 | 49.4% | 186 | 50.5% | 66 | 25.4% |
| **25.1.1. Do you boil this milk? (Choose One)** | No | 281 | 52.9% | 71 | 45.5% | 96 | 53.0% | 114 | 58.8% |
|  | Yes | 250 | 47.1% | 85 | 54.5% | 85 | 47.0% | 80 | 41.2% |
| **25.2. Do you or your family ever drink milk from other people’s animals? (Choose One)** | Yes | 608 | 57.5% | 176 | 45.8% | 247 | 66.6% | 185 | 61.3% |
|  | No | 449 | 42.5% | 208 | 54.2% | 124 | 33.4% | 117 | 38.7% |
| **24.2.1. Where do you normally buy milk? (Choose All That Apply)** | Farm | 427 | 70.6% | 113 | 64.6% | 183 | 74.4% | 131 | 71.2% |
|  | Market | 292 | 48.3% | 67 | 38.3% | 141 | 57.3% | 84 | 45.7% |
|  | Supermarket | 0 | 0.0% | 0 | 0.0% | 0 | 0.0% | 0 | 0.0% |
| **25.3. Do you have access to commercial milk? (Choose One)** | No | 678 | 64.4% | 207 | 53.9% | 239 | 65.1% | 232 | 76.8% |
|  | Yes | 375 | 35.6% | 177 | 46.1% | 128 | 34.9% | 70 | 23.2% |
| **25.3.1. Do you or your family drink this commercial milk? (Choose One)** | Yes | 311 | 82.9% | 156 | 88.1% | 106 | 82.8% | 49 | 70.0% |
|  | No | 64 | 17.1% | 21 | 11.9% | 22 | 17.2% | 21 | 30.0% |
| **26. Do you or your family ever drink or eat products made from milk? (Choose One)** | No | 1,143 | 94.2% | 440 | 96.9% | 334 | 87.4% | 369 | 97.6% |
|  | Yes | 71 | 5.8% | 14 | 3.1% | 48 | 12.6% | 9 | 2.4% |
| **26.1. What products do you purchase to eat or drink? (Enter Products)** | Cheese | 45 | 66.2% | 14 | 100% | 24 | 52.2% | 7 | 87.5% |
|  | Yogurt | 39 | 57.4% | 0 | 0.0% | 39 | 84.8% | 0 | 0.0% |
|  | Butter | 2 | 2.9% | 0 | 0.0% | 1 | 2.2% | 1 | 12.5% |
| **26.2. Do you or your family produce these products from milk that comes from your animals? (Choose One)** | No | 61 | 87.1% | 9 | 64.3% | 47 | 97.9% | 5 | 55.6% |
|  | Yes | 9 | 12.9% | 5 | 35.7% | 1 | 2.1% | 4 | 44.4% |
| **26.2.1 Do you boil the milk before you make these products? (Choose One)** | Yes | 9 | 100% | 5 | 100% | 1 | 100% | 4 | 100% |

**Supplementary Table 2: Cameroonian livestock trader survey questions and results**

|  | | **Countrywide** | | **Far North** | | **North** | | **West** | |
| --- | --- | --- | --- | --- | --- | --- | --- | --- | --- |
| **Survey Question** | **Answer** | **Frequency │ Rate (%)** | | **Frequency │ Rate (%)** | | **Frequency │ Rate (%)** | | **Frequency │ Rate (%)** | |
| **Number of Livestock Traders** | Livestock Traders | 1,147 | 100% | 385 | 100% | 374 | 100.0% | 388 | 100% |
| **1. Gender** | Male | 1,132 | 98.7% | 381 | 99.0% | 363 | 97.1% | 388 | 100% |
|  | Female | 15 | 1.3% | 4 | 1.0% | 11 | 2.9% | 0 | 0.0% |
| **2. What is your age? (Enter a Number)** | 18-27 | 6 | 0.5% | 1 | 0.3% | 5 | 1.3% | 0 | 0.0% |
|  | 28-37 | 127 | 11.1% | 61 | 15.8% | 38 | 10.2% | 28 | 7.2% |
|  | 38-47 | 633 | 55.2% | 256 | 66.5% | 125 | 33.4% | 252 | 64.9% |
|  | 48-57 | 328 | 28.6% | 50 | 13.0% | 184 | 49.2% | 94 | 24.2% |
|  | 58-67 | 43 | 3.7% | 9 | 2.3% | 22 | 5.9% | 12 | 3.1% |
|  | 68+ | 10 | 0.9% | 8 | 2.1% | 0 | 0.0% | 2 | 0.5% |
| **3. Which ethnic group do you identify with? (Enter Group)** | Peul | 297 | 25.9% | 52 | 13.5% | 129 | 34.6% | 116 | 29.9% |
|  | Bamum | 168 | 14.7% | 1 | 0.3% | 1 | 0.3% | 166 | 42.8% |
|  | Masa | 84 | 7.3% | 61 | 15.9% | 23 | 6.2% | 0 | 0.0% |
|  | Bamiléké | 79 | 6.9% | 4 | 1.0% | 18 | 4.8% | 57 | 14.7% |
|  | Laka | 55 | 4.8% | 2 | 0.5% | 53 | 14.2% | 0 | 0.0% |
|  | Tupuri | 55 | 4.8% | 17 | 4.4% | 38 | 10.2% | 0 | 0.0% |
|  | Musgum | 47 | 4.1% | 47 | 12.2% | 0 | 0.0% | 0 | 0.0% |
|  | Fali | 38 | 3.3% | 13 | 3.4% | 24 | 6.4% | 1 | 0.3% |
|  | Guiziga | 34 | 3.0% | 13 | 3.4% | 21 | 5.6% | 0 | 0.0% |
|  | Kotoko | 30 | 2.6% | 30 | 7.8% | 0 | 0.0% | 0 | 0.0% |
|  | Choa-Arab | 28 | 2.4% | 28 | 7.3% | 0 | 0.0% | 0 | 0.0% |
|  | Haousa | 27 | 2.4% | 0 | 0.0% | 10 | 2.7% | 17 | 4.4% |
|  | Mofu | 26 | 2.3% | 25 | 6.5% | 1 | 0.3% | 0 | 0.0% |
|  | Mundang | 23 | 2.0% | 23 | 6.0% | 0 | 0.0% | 0 | 0.0% |
|  | Mbum | 22 | 1.9% | 1 | 0.3% | 21 | 5.6% | 0 | 0.0% |
|  | Other (49 different groups) | 132 | 11.5% | 67 | 17.4% | 34 | 9.1% | 31 | 8.0% |
| **4. Do you know how to read? (Choose One)** | No | 613 | 54.8% | 212 | 55.1% | 172 | 49.9% | 229 | 59.0% |
|  | Yes | 505 | 45.2% | 173 | 44.9% | 173 | 50.1% | 159 | 41.0% |
| **5. What is your highest level of education? (Choose One)** | No formal education | 525 | 46.0% | 146 | 37.9% | 215 | 58.3% | 164 | 42.3% |
|  | Primary school | 249 | 21.8% | 91 | 23.6% | 58 | 15.7% | 100 | 25.8% |
|  | Secondary school | 285 | 25.0% | 96 | 24.9% | 79 | 21.4% | 110 | 28.4% |
|  | Technical School | 56 | 4.9% | 45 | 11.7% | 0 | 0.0% | 11 | 2.8% |
|  | University | 27 | 2.4% | 7 | 1.8% | 17 | 4.6% | 3 | 0.8% |
| **6. Which animals do you buy and sell? (Choose All That Apply)** | Goats | 573 | 50.0% | 245 | 63.6% | 122 | 32.6% | 206 | 53.1% |
|  | Sheep | 542 | 47.3% | 217 | 56.4% | 118 | 31.6% | 207 | 53.4% |
|  | Cattle | 479 | 41.8% | 164 | 42.6% | 145 | 38.8% | 170 | 43.8% |
|  | Pigs | 125 | 10.9% | 47 | 12.2% | 49 | 13.1% | 29 | 7.5% |
| **7. What is the purpose of the livestock that you purchase? (Choose All That Apply)** | Live trade | 663 | 57.8% | 291 | 75.6% | 8 | 2.1% | 364 | 93.8% |
|  | Slaughter | 533 | 46.5% | 96 | 24.9% | 371 | 99.2% | 66 | 17.0% |
| **8. When purchasing cattle, how many of these animals do you usually purchase in a single day? (Enter a Number)** | 0 | 658 | 57.4% | 214 | 55.6% | 235 | 62.8% | 209 | 53.9% |
|  | 1-5 | 382 | 33.3% | 171 | 44.4% | 69 | 18.4% | 142 | 36.6% |
|  | 6-10 | 92 | 8.0% | 0 | 0.0% | 57 | 15.2% | 35 | 9.0% |
|  | 11+ | 15 | 1.3% | 0 | 0.0% | 13 | 3.5% | 2 | 0.5% |
| **9. When purchasing goats, how many of these animals do you usually purchase in a single day? (Enter a Number)** | 0 | 532 | 46.4% | 129 | 33.5% | 235 | 62.8% | 168 | 43.3% |
|  | 1-5 | 527 | 45.9% | 256 | 66.5% | 84 | 22.5% | 187 | 48.2% |
|  | 6-10 | 75 | 6.5% | 0 | 0.0% | 44 | 11.8% | 31 | 8.0% |
|  | 11+ | 13 | 1.1% | 0 | 0.0% | 11 | 2.9% | 2 | 0.5% |
| **10. When purchasing sheep, how many of these animals do you usually purchase in a single day? (Enter a Number)** | 0 | 577 | 50.3% | 167 | 43.4% | 230 | 61.5% | 180 | 46.4% |
|  | 1-5 | 511 | 44.6% | 218 | 56.6% | 114 | 30.5% | 179 | 46.1% |
|  | 6-10 | 50 | 4.4% | 0 | 0.0% | 22 | 5.9% | 28 | 7.2% |
|  | 11+ | 9 | 0.8% | 0 | 0.0% | 8 | 2.1% | 1 | 0.3% |
| **11. When purchasing pigs, how many of these animals do you usually purchase in a single day? (Enter a Number)** | 0 | 1,021 | 89.0% | 337 | 87.5% | 325 | 86.9% | 359 | 92.5% |
|  | 1-5 | 114 | 9.9% | 48 | 12.5% | 38 | 10.2% | 28 | 7.2% |
|  | 6-10 | 11 | 1.0% | 0 | 0.0% | 11 | 2.9% | 0 | 0.0% |
|  | 11+ | 1 | 0.1% | 0 | 0.0% | 0 | 0.0% | 1 | 0.3% |
| **12. Ordinarily, how often do you acquire new livestock? (Choose One)** | Never | 0 | 0.0% | 0 | 0.0% | 0 | 0.0% | 0 | 0.0% |
|  | Daily | 364 | 31.8% | 111 | 29.0% | 193 | 51.9% | 60 | 15.5% |
|  | Weekly | 681 | 59.6% | 233 | 60.8% | 162 | 43.5% | 286 | 73.7% |
|  | Monthly | 73 | 6.4% | 21 | 5.5% | 16 | 4.3% | 36 | 9.3% |
|  | Yearly | 1 | 0.1% | 0 | 0.0% | 1 | 0.3% | 0 | 0.0% |
|  | As needed | 24 | 2.1% | 18 | 4.7% | 0 | 0.0% | 6 | 1.5% |
| **13. From which of these locations do you typically purchase livestock? (Choose All That Apply)** | Market | 1,053 | 92.0% | 357 | 93.0% | 333 | 89.3% | 363 | 93.6% |
|  | Farm | 347 | 30.3% | 46 | 12.0% | 230 | 61.7% | 71 | 18.3% |
| **14. How many different farms and markets do you frequently visit to purchase livestock? (Enter a Number)** | 1 | 429 | 37.5% | 158 | 41.1% | 138 | 37.2% | 133 | 34.3% |
|  | 2 | 442 | 38.7% | 161 | 41.9% | 111 | 29.9% | 170 | 43.8% |
|  | 3 | 215 | 18.8% | 50 | 13.0% | 95 | 25.6% | 70 | 18.0% |
|  | 4 | 45 | 3.9% | 9 | 2.3% | 25 | 6.7% | 11 | 2.8% |
|  | 5+ | 12 | 1.0% | 6 | 1.6% | 2 | 0.5% | 4 | 1.0% |
| **15. Where are the livestock normally kept before you purchase them? (Choose All That Apply)** | Open pasture that belongs to someone else | 663 | 58.9% | 145 | 37.9% | 216 | 58.1% | 302 | 81.6% |
|  | Fenced area that belongs to someone else | 329 | 29.2% | 117 | 30.5% | 164 | 44.1% | 48 | 13.0% |
|  | Livestock pen that belongs to someone else | 278 | 24.7% | 119 | 31.1% | 123 | 33.1% | 36 | 9.7% |
|  | Livestock pen that belongs to you | 52 | 4.6% | 1 | 0.3% | 49 | 13.2% | 2 | 0.5% |
|  | Open pasture that belongs to you | 15 | 1.3% | 0 | 0.0% | 14 | 3.8% | 1 | 0.3% |
|  | Fenced area that belongs to you | 12 | 1.1% | 1 | 0.3% | 11 | 3.0% | 0 | 0.0% |
| **16. Where do you normally keep your livestock before you sell or slaughter them? (Choose All That Apply)** | Livestock pen that belongs to someone else | 561 | 49.2% | 100 | 26.0% | 236 | 63.6% | 225 | 58.3% |
|  | Livestock pen that belongs to you | 398 | 34.9% | 197 | 51.3% | 84 | 22.6% | 117 | 30.3% |
|  | Fenced area that belongs to someone else | 187 | 16.4% | 16 | 4.2% | 126 | 34.0% | 45 | 11.7% |
|  | Open pasture that belongs to someone else | 142 | 12.4% | 0 | 0.0% | 142 | 38.3% | 0 | 0.0% |
|  | Fenced area that belongs to you | 81 | 7.1% | 71 | 18.5% | 8 | 2.2% | 2 | 0.5% |
|  | Open pasture that belongs to you | 6 | 0.5% | 0 | 0.0% | 6 | 1.6% | 0 | 0.0% |
| **17. Do you keep your livestock along with animals that you do not own? (Choose One)** | Yes | 623 | 54.8% | 214 | 55.6% | 126 | 34.6% | 283 | 72.9% |
|  | No | 514 | 45.2% | 171 | 44.4% | 238 | 65.4% | 105 | 27.1% |
| **18. How do you usually transport your livestock? (Choose All That Apply)** | Walk | 764 | 67.6% | 182 | 49.2% | 331 | 88.7% | 251 | 64.7% |
|  | Motorcycle | 544 | 48.1% | 130 | 35.1% | 276 | 74.0% | 138 | 35.6% |
|  | Truck | 157 | 13.9% | 69 | 18.6% | 60 | 16.1% | 28 | 7.2% |
| **19. How many days do you generally keep livestock before sale or slaughter? (Enter a Number)** | 0 | 4 | 0.3% | 2 | 0.5% | 0 | 0.0% | 2 | 0.5% |
|  | 1-5 | 483 | 42.1% | 188 | 48.8% | 179 | 47.9% | 116 | 29.9% |
|  | 6-10 | 291 | 25.4% | 35 | 9.1% | 190 | 50.8% | 66 | 17.0% |
|  | 11-15 | 165 | 14.4% | 47 | 12.2% | 3 | 0.8% | 115 | 29.6% |
|  | 16-20 | 7 | 0.6% | 4 | 1.0% | 1 | 0.3% | 2 | 0.5% |
|  | 21-25 | 69 | 6.0% | 9 | 2.3% | 0 | 0.0% | 60 | 15.5% |
|  | 26-30 | 48 | 4.2% | 24 | 6.2% | 1 | 0.3% | 23 | 5.9% |
|  | 31+ | 80 | 7.0% | 76 | 19.7% | 0 | 0.0% | 4 | 1.0% |
| **20. Normally, how often do you sell or slaughter livestock? (Choose One)** | Daily | 476 | 42.1% | 126 | 33.3% | 262 | 70.1% | 88 | 23.2% |
|  | Weekly | 380 | 33.6% | 124 | 32.8% | 95 | 25.4% | 161 | 42.5% |
|  | Monthly | 250 | 22.1% | 107 | 28.3% | 15 | 4.0% | 128 | 33.8% |
|  | Yearly | 3 | 0.3% | 2 | 0.5% | 1 | 0.3% | 0 | 0.0% |
|  | As needed | 22 | 1.9% | 19 | 5.0% | 1 | 0.3% | 2 | 0.5% |
| **21. What do you ordinarily do with meat after your livestock have been slaughtered? (Choose All That Apply)** | Take it to a market to sell to a person for their food | 465 | 64.2% | 90 | 90.9% | 136 | 36.4% | 239 | 95.2% |
|  | Sell it at the slaughter facility to a person for their food | 288 | 39.8% | 17 | 17.2% | 229 | 61.2% | 42 | 16.7% |
|  | Sell it at the slaughter facility to a market shop owner | 288 | 39.8% | 1 | 1.0% | 278 | 74.3% | 9 | 3.6% |
|  | Take it home to eat | 124 | 17.1% | 0 | 0.0% | 57 | 15.2% | 67 | 26.7% |
|  | Take it to a market to sell to a shop owner | 38 | 5.2% | 1 | 1.0% | 34 | 9.1% | 3 | 1.2% |
| **22. What do you ordinarily do with the offal after your livestock have been slaughtered? (Choose All That Apply)** | Take it home to eat | 397 | 54.9% | 21 | 21.4% | 194 | 52.0% | 182 | 72.2% |
|  | Take it to a market to sell to a person for their food | 346 | 47.9% | 72 | 73.5% | 133 | 35.7% | 141 | 56.0% |
|  | Sell it at the slaughter facility to a person for their food | 277 | 38.3% | 14 | 14.3% | 231 | 61.9% | 32 | 12.7% |
|  | Sell it at the slaughter facility to a market shop owner | 276 | 38.2% | 0 | 0.0% | 268 | 71.8% | 8 | 3.2% |
|  | Take it to a market to sell to a shop owner | 44 | 6.1% | 0 | 0.0% | 41 | 11.0% | 3 | 1.2% |
| **23. Do you always wear boots to farms, markets, and slaughter facilities? (Choose One)** | No | 1,047 | 92.7% | 382 | 99.2% | 286 | 79.4% | 379 | 98.7% |
|  | Yes | 82 | 7.3% | 3 | 0.8% | 74 | 20.6% | 5 | 1.3% |
| **23.1. Do you always wash your boots with soap and water before entering and before leaving farms, markets, and slaughter facilities? (Choose One)** | Yes | 59 | 73.8% | 0 | 0.0% | 55 | 76.4% | 4 | 80.0% |
|  | No | 21 | 26.3% | 3 | 100% | 17 | 23.6% | 1 | 20.0% |
| **24. Do you always wash your hands with soap and water before entering and before leaving farms, markets, and slaughter facilities? (Choose One)** | No | 1,015 | 90.2% | 372 | 96.6% | 284 | 80.2% | 359 | 93.0% |
|  | Yes | 110 | 9.8% | 13 | 3.4% | 70 | 19.8% | 27 | 7.0% |
| **25. Can drinking raw (unboiled) milk spread certain animal diseases to people? (Choose One)** | I do not know | 742 | 66.8% | 270 | 70.1% | 223 | 65.8% | 249 | 64.5% |
|  | No | 335 | 30.2% | 112 | 29.1% | 92 | 27.1% | 131 | 33.9% |
|  | Yes | 33 | 3.0% | 3 | 0.8% | 24 | 7.1% | 6 | 1.6% |

**Supplementary Table 3: Cameroonian slaughter facility inspector survey questions and results**

|  | | **Countrywide** | | **Far North** | | **North** | | **West** | |
| --- | --- | --- | --- | --- | --- | --- | --- | --- | --- |
| **Survey Question** | **Answer** | **Frequency │ Rate (%)** | | **Frequency │ Rate (%)** | | **Frequency │ Rate (%)** | | **Frequency │ Rate (%)** | |
| **Number of inspectors** | Inspectors | 36 | 100% | 10 | 100% | 12 | 100% | 14 | 100% |
| **1. Gender (Choose One)** | Male | 19 | 52.8% | 6 | 60.0% | 7 | 58.3% | 6 | 42.9% |
|  | Female | 17 | 47.2% | 4 | 40.0% | 5 | 41.7% | 8 | 57.1% |
| **2. How many years have you been an inspector? (Enter a Number)** | <5 | 13 | 36.1% | 5 | 50.0% | 4 | 33.3% | 4 | 28.6% |
|  | 5-9 | 10 | 27.8% | 1 | 10.0% | 6 | 50.0% | 3 | 21.4% |
|  | 10-14 | 7 | 19.4% | 1 | 10.0% | 2 | 16.7% | 4 | 28.6% |
|  | 15-19 | 3 | 8.3% | 1 | 10.0% | 0 | 0.0% | 2 | 14.3% |
|  | 20-25 | 3 | 8.3% | 2 | 20.0% | 0 | 0.0% | 1 | 7.1% |
| **3. Which of these animals are slaughtered at this facility? (Choose All That Apply)** | Cattle | 24 | 66.7% | 3 | 30.0% | 11 | 91.7% | 10 | 71.4% |
|  | Goats | 12 | 33.3% | 8 | 80.0% | 4 | 33.3% | 0 | 0.0% |
|  | Sheep | 11 | 30.6% | 8 | 80.0% | 3 | 25.0% | 0 | 0.0% |
|  | Pigs | 7 | 19.4% | 1 | 10.0% | 1 | 8.3% | 5 | 35.7% |
| **4. Are all of the livestock at this facility inspected for disease before slaughter? (Choose One)** | No | 18 | 50.0% | 5 | 50.0% | 2 | 16.7% | 11 | 78.6% |
|  | Yes | 18 | 50.0% | 5 | 50.0% | 10 | 83.3% | 3 | 21.4% |
| **5. Are all of the livestock at this facility inspected for disease during slaughter? (Choose One)** | Yes | 26 | 72.2% | 5 | 50.0% | 8 | 66.7% | 13 | 92.9% |
|  | No | 10 | 27.8% | 5 | 50.0% | 4 | 33.3% | 1 | 7.1% |
| **6. How many animals do you generally inspect per day? (Enter a Number)** | <10 | 14 | 45.2% | 5 | 50.0% | 5 | 41.7% | 4 | 28.6% |
|  | 10-19 | 0 | 0.0% | 0 | 0.0% | 0 | 0.0% | 0 | 0.0% |
|  | 20-29 | 4 | 12.9% | 2 | 20.0% | 0 | 0.0% | 2 | 14.3% |
|  | 30-39 | 2 | 6.5% | 1 | 10.0% | 0 | 0.0% | 6 | 42.9% |
|  | 40-49 | 1 | 3.2% | 0 | 0.0% | 0 | 0.0% | 1 | 7.1% |
|  | 50+ | 10 | 32.3% | 2 | 20.0% | 7 | 58.3% | 1 | 7.1% |
| **7. Have you heard of brucellosis (baakaale)? (Check one)** | No | 2 | 5.6% | 1 | 10.0% | 0 | 0.0% | 1 | 7.1% |
|  | Yes | 34 | 94.4% | 9 | 90.0% | 12 | 100.0% | 13 | 92.9% |
| **8. Which of these are possible symptoms of brucellosis in cattle? (Choose All That Apply)** | Abortion | 29 | 82.9% | 9 | 90.0% | 9 | 81.8% | 11 | 78.6% |
|  | Hygromas | 24 | 68.6% | 7 | 70.0% | 10 | 90.9% | 7 | 50.0% |
|  | Reduced fertility | 22 | 62.9% | 3 | 30.0% | 10 | 90.9% | 9 | 64.3% |
|  | Swollen/inflamed testicle | 13 | 37.1% | 1 | 10.0% | 6 | 54.5% | 6 | 42.9% |
|  | Bloody manure/feces | 5 | 14.3% | 0 | 0.0% | 3 | 27.3% | 2 | 14.3% |
|  | Difficult breathing | 3 | 8.6% | 0 | 0.0% | 0 | 0.0% | 3 | 21.4% |
|  | Red eyes with tears | 3 | 8.6% | 0 | 0.0% | 1 | 9.1% | 2 | 14.3% |
|  | Skin rash | 3 | 8.6% | 0 | 0.0% | 1 | 9.1% | 2 | 14.3% |
|  | Do not know | 2 | 5.7% | 0 | 0.0% | 0 | 0.0% | 2 | 14.3% |
| **9. Which of these are possible symptoms of brucellosis in sheep and goats? (Choose All That Apply)** | Abortion | 27 | 79.4% | 7 | 20.6% | 11 | 91.7% | 9 | 75.0% |
|  | Reduced fertility | 20 | 58.8% | 2 | 5.9% | 10 | 83.3% | 8 | 66.7% |
|  | Swollen/inflamed testicle | 13 | 38.2% | 1 | 2.9% | 6 | 50.0% | 6 | 50.0% |
|  | Hygromas | 12 | 35.3% | 0 | 0.0% | 10 | 83.3% | 2 | 16.7% |
|  | Do not know | 5 | 14.7% | 2 | 5.9% | 0 | 0.0% | 3 | 25.0% |
|  | Red eyes with tears | 4 | 11.8% | 0 | 0.0% | 3 | 25.0% | 1 | 8.3% |
|  | Bloody manure/feces | 2 | 5.9% | 0 | 0.0% | 1 | 8.3% | 1 | 8.3% |
|  | Difficult breathing | 2 | 5.9% | 0 | 0.0% | 1 | 8.3% | 1 | 8.3% |
|  | Skin rash | 2 | 5.9% | 0 | 0.0% | 1 | 8.3% | 1 | 8.3% |
|  | There are no signs | 1 | 2.9% | 1 | 2.9% | 0 | 0.0% | 0 | 0.0% |
| **10. Which of these are possible symptoms of brucellosis in swine? (Choose All That Apply)** | Abortion | 22 | 64.7% | 2 | 20.0% | 11 | 91.7% | 10 | 76.9% |
|  | Reduced fertility | 19 | 55.9% | 1 | 10.0% | 10 | 83.3% | 8 | 61.5% |
|  | Hygromas | 12 | 35.3% | 0 | 0.0% | 11 | 91.7% | 1 | 7.7% |
|  | Swollen/inflamed testicle | 12 | 35.3% | 0 | 0.0% | 6 | 50.0% | 6 | 46.2% |
|  | Do not know | 10 | 29.4% | 7 | 70.0% | 0 | 0.0% | 3 | 23.1% |
|  | Red eyes with tears | 4 | 11.8% | 0 | 0.0% | 3 | 25.0% | 1 | 7.7% |
|  | Bloody manure/feces | 3 | 8.8% | 0 | 0.0% | 2 | 16.7% | 1 | 7.7% |
|  | Difficult breathing | 2 | 5.9% | 0 | 0.0% | 1 | 8.3% | 1 | 7.7% |
|  | Skin rash | 2 | 5.9% | 0 | 0.0% | 1 | 8.3% | 1 | 7.7% |
| **11. Which of these are possible signs of brucellosis in slaughtered cattle? (Choose All That Apply)** | Enlarged lymph nodes | 13 | 38.2% | 0 | 0.0% | 10 | 83.3% | 3 | 25.0% |
|  | Placenta is thickened, yellow-grey, and may have pus on surface | 12 | 35.3% | 0 | 0.0% | 8 | 66.7% | 4 | 33.3% |
|  | Do not know | 9 | 26.5% | 5 | 50.0% | 0 | 0.0% | 4 | 33.3% |
|  | Blood or pus in the uterus | 8 | 23.5% | 0 | 0.0% | 4 | 33.3% | 4 | 33.3% |
|  | Swollen or dark mammary glands/udders | 8 | 23.5% | 0 | 0.0% | 4 | 33.3% | 4 | 33.3% |
|  | Widespread bleeding over organ and body surfaces | 6 | 17.6% | 0 | 0.0% | 4 | 33.3% | 2 | 16.7% |
|  | There are no signs | 5 | 14.7% | 5 | 50.0% | 0 | 0.0% | 0 | 0.0% |
|  | Pinpoint spots of blood on the surface of the kidneys | 3 | 8.8% | 0 | 0.0% | 3 | 25.0% | 0 | 0.0% |
|  | Dark tarry blood that does not clot properly | 2 | 5.9% | 0 | 0.0% | 1 | 8.3% | 1 | 8.3% |
|  | Pale or dark red spots in the heart | 2 | 5.9% | 0 | 0.0% | 1 | 8.3% | 1 | 8.3% |
| **12. Which of these are possible signs of brucellosis in slaughtered sheep and goats? (Choose All That Apply)** | Enlarged lymph nodes | 12 | 36.4% | 0 | 0.0% | 10 | 83.3% | 2 | 18.2% |
|  | Do not know | 10 | 30.3% | 5 | 50.0% | 0 | 0.0% | 5 | 45.5% |
|  | Placenta is thickened, yellow-grey, and may have pus on surface | 10 | 30.3% | 0 | 0.0% | 6 | 50.0% | 4 | 36.4% |
|  | Swollen or dark mammary glands/udders | 8 | 24.2% | 0 | 0.0% | 5 | 41.7% | 3 | 27.3% |
|  | Blood or pus in the uterus | 7 | 21.2% | 0 | 0.0% | 4 | 33.3% | 3 | 27.3% |
|  | There are no signs | 5 | 15.2% | 5 | 50.0% | 0 | 0.0% | 0 | 0.0% |
|  | Widespread bleeding over organ and body surfaces | 5 | 15.2% | 0 | 0.0% | 4 | 33.3% | 1 | 9.1% |
|  | Dark tarry blood that does not clot properly | 2 | 6.1% | 0 | 0.0% | 1 | 8.3% | 1 | 9.1% |
|  | Pale or dark red spots in the heart | 2 | 6.1% | 0 | 0.0% | 1 | 8.3% | 1 | 9.1% |
|  | Pinpoint spots of blood on the surface of the kidneys | 2 | 6.1% | 0 | 0.0% | 2 | 16.7% | 0 | 0.0% |
| **13. Which of these are possible signs of brucellosis in slaughtered in swine? (Choose All That Apply)** | Do not know | 13 | 38.2% | 8 | 80.0% | 0 | 0.0% | 5 | 41.7% |
|  | Placenta is thickened, yellow-grey, and may have pus on surface | 13 | 38.2% | 0 | 0.0% | 8 | 66.7% | 5 | 41.7% |
|  | Enlarged lymph nodes | 12 | 35.3% | 0 | 0.0% | 9 | 75.0% | 3 | 25.0% |
|  | Blood or pus in the uterus | 8 | 23.5% | 0 | 0.0% | 4 | 33.3% | 4 | 33.3% |
|  | Swollen or dark mammary glands/udders | 8 | 23.5% | 0 | 0.0% | 4 | 33.3% | 4 | 33.3% |
|  | Widespread bleeding over organ and body surfaces | 6 | 17.6% | 0 | 0.0% | 4 | 33.3% | 2 | 16.7% |
|  | Pinpoint spots of blood on the surface of the kidneys | 3 | 8.8% | 0 | 0.0% | 2 | 16.7% | 1 | 8.3% |
|  | Dark tarry blood that does not clot properly | 2 | 5.9% | 0 | 0.0% | 1 | 8.3% | 1 | 8.3% |
|  | Pale or dark red spots in the heart | 2 | 5.9% | 0 | 0.0% | 1 | 8.3% | 1 | 8.3% |
|  | There are no signs | 2 | 5.9% | 2 | 20.0% | 0 | 0.0% | 0 | 0.0% |
| **14. Which of these diseases may look similar to brucellosis in cattle? (Choose All That Apply)** | Do not know | 15 | 45.5% | 6 | 60.0% | 4 | 36.4% | 5 | 41.7% |
|  | There is no differential diagnosis | 7 | 21.2% | 2 | 20.0% | 4 | 36.4% | 1 | 8.3% |
|  | Contagious bovine pleuropneumonia (CBPP) | 6 | 18.2% | 1 | 10.0% | 0 | 0.0% | 5 | 41.7% |
|  | Trichomoniasis | 5 | 15.2% | 0 | 0.0% | 2 | 18.2% | 3 | 25.0% |
|  | Trypanosomiasis | 3 | 9.1% | 1 | 10.0% | 1 | 9.1% | 1 | 8.3% |
|  | Foot-and-mouth disease (FMD) | 2 | 6.1% | 0 | 0.0% | 0 | 0.0% | 2 | 16.7% |
|  | Leptospirosis | 2 | 6.1% | 0 | 0.0% | 0 | 0.0% | 2 | 16.7% |
|  | Vibriosis | 1 | 3.0% | 0 | 0.0% | 0 | 0.0% | 1 | 8.3% |
|  | Infectious bovine rhinotracheitis | 0 | 0.0% | 0 | 0.0% | 0 | 0.0% | 0 | 0.0% |
|  | Listeriosis | 0 | 0.0% | 0 | 0.0% | 0 | 0.0% | 0 | 0.0% |
|  | Mycoses | 0 | 0.0% | 0 | 0.0% | 0 | 0.0% | 0 | 0.0% |
| **15. Which of these diseases may look similar to brucellosis in sheep and goats? (Choose All That Apply)** | Do not know | 19 | 55.9% | 9 | 90.0% | 4 | 36.4% | 6 | 46.2% |
|  | There is no differential diagnosis | 7 | 20.6% | 1 | 10.0% | 5 | 45.5% | 1 | 7.7% |
|  | Chlamydiosis | 5 | 14.7% | 0 | 0.0% | 1 | 9.1% | 4 | 30.8% |
|  | Contagious caprine pleuropneumonia (CCPP) | 3 | 8.8% | 0 | 0.0% | 0 | 0.0% | 3 | 23.1% |
|  | Foot-and-mouth disease (FMD) | 1 | 2.9% | 0 | 0.0% | 0 | 0.0% | 1 | 7.7% |
|  | Trypanosomiasis | 1 | 2.9% | 0 | 0.0% | 1 | 9.1% | 0 | 0.0% |
|  | Coxiellosis | 0 | 0.0% | 0 | 0.0% | 0 | 0.0% | 0 | 0.0% |
|  | Peste des petits ruminants (PPR) | 0 | 0.0% | 0 | 0.0% | 0 | 0.0% | 0 | 0.0% |
| **16. Which of these diseases may look similar to brucellosis in pigs? (Choose All That Apply)** | Do not know | 18 | 52.9% | 8 | 80.0% | 4 | 36.4% | 6 | 46.2% |
|  | There is no differential diagnosis | 9 | 26.5% | 2 | 20.0% | 6 | 54.5% | 1 | 7.7% |
|  | Salmonellosis | 5 | 14.7% | 0 | 0.0% | 1 | 9.1% | 4 | 30.8% |
|  | African swine fever (ASF) | 2 | 5.9% | 0 | 0.0% | 0 | 0.0% | 2 | 15.4% |
|  | Leptospirosis | 2 | 5.9% | 0 | 0.0% | 0 | 0.0% | 2 | 15.4% |
|  | Streptococcidiosis | 2 | 5.9% | 0 | 0.0% | 1 | 9.1% | 1 | 7.7% |
|  | Foot-and-mouth disease (FMD) | 1 | 2.9% | 0 | 0.0% | 0 | 0.0% | 1 | 7.7% |
|  | Classical swine fever (CSF) | 0 | 0.0% | 0 | 0.0% | 0 | 0.0% | 0 | 0.0% |
|  | Clostridium difficile enteritis | 0 | 0.0% | 0 | 0.0% | 0 | 0.0% | 0 | 0.0% |
|  | Parvovirosis | 0 | 0.0% | 0 | 0.0% | 0 | 0.0% | 0 | 0.0% |
| **17. How often do you identify possible cases of brucellosis in live animals? (Choose One)** | Daily | 1 | 2.8% | 0 | 0.0% | 1 | 8.3% | 0 | 0.0% |
|  | Weekly | 1 | 2.8% | 1 | 10.0% | 0 | 0.0% | 0 | 0.0% |
|  | Monthly | 3 | 8.3% | 1 | 10.0% | 1 | 8.3% | 1 | 7.1% |
|  | Yearly | 8 | 22.2% | 3 | 30.0% | 4 | 33.3% | 1 | 7.1% |
|  | Seasonally | 3 | 8.3% | 0 | 0.0% | 3 | 25.0% | 0 | 0.0% |
|  | No Answer | 20 | 55.6% | 5 | 50.0% | 3 | 25.0% | 12 | 85.7% |
| **18. How often do you identify possible cases of brucellosis in slaughtered animals? (Choose One)** | Daily | 0 | 0.0% | 0 | 0.0% | 0 | 0.0% | 0 | 0.0% |
|  | Weekly | 1 | 2.8% | 0 | 0.0% | 0 | 0.0% | 0 | 0.0% |
|  | Monthly | 2 | 5.6% | 0 | 0.0% | 2 | 16.7% | 0 | 0.0% |
|  | Yearly | 6 | 16.7% | 1 | 10.0% | 4 | 33.3% | 2 | 14.3% |
|  | Seasonally | 2 | 5.6% | 0 | 0.0% | 2 | 16.7% | 0 | 0.0% |
|  | No Answer | 25 | 69.4% | 9 | 90.0% | 4 | 33.3% | 12 | 85.7% |
| **19. What do you do if you believe a live animal has brucellosis? (Choose One)** | Allow the animal to be slaughtered | 16 | 44.4% | 6 | 60.0% | 7 | 58.3% | 3 | 21.4% |
|  | Reject the animal from being slaughtered | 11 | 30.6% | 3 | 30.0% | 4 | 33.3% | 4 | 28.6% |
|  | No Answer | 9 | 25.0% | 1 | 10.0% | 1 | 8.3% | 7 | 50.0% |
| **20. What do you do if you believe a slaughtered animal has brucellosis? (Choose One)** | Condemn the part with lesions | 20 | 55.6% | 2 | 20.0% | 9 | 75.0% | 9 | 64.3% |
|  | Allow the entire animal to be processed | 6 | 16.7% | 6 | 60.0% | 0 | 0.0% | 0 | 0.0% |
|  | No Answer | 6 | 16.7% | 2 | 20.0% | 0 | 0.0% | 4 | 28.6% |
|  | Condemn the entire animal | 4 | 11.1% | 0 | 0.0% | 3 | 25.0% | 1 | 7.1% |
| **21. If you believe an animal has brucellosis, do you call LANAVET? (Choose One)** | No | 18 | 50.0% | 8 | 80.0% | 3 | 25.0% | 7 | 50.0% |
|  | Yes | 13 | 36.1% | 2 | 20.0% | 7 | 58.3% | 4 | 28.6% |
|  | No Answer | 5 | 13.9% | 0 | 0.0% | 2 | 16.7% | 3 | 21.4% |
| **22. When a slaughtered animal is condemned due to disease, what happens to it? (Choose All That Apply)** | Buried | 23 | 63.9% | 6 | 60.0% | 10 | 83.3% | 7 | 50.0% |
|  | Burned | 21 | 58.3% | 3 | 30.0% | 5 | 41.7% | 13 | 92.9% |
|  | Given to dogs or wild animals | 1 | 2.8% | 1 | 10.0% | 0 | 0.0% | 0 | 0.0% |
| **23. During slaughter, what happens to the fetus and placenta of a pregnant animal? (Choose All That Apply)** | Buried | 15 | 46.9% | 3 | 33.3% | 11 | 91.7% | 1 | 9.1% |
|  | Given to dogs or wild animals | 15 | 46.9% | 5 | 55.6% | 0 | 0.0% | 10 | 90.9% |
|  | Burned | 7 | 21.9% | 2 | 22.2% | 4 | 33.3% | 1 | 9.1% |
| **24. While working at a slaughter facility, do you always wear rubber boots? (Choose One)** | Yes | 29 | 80.6% | 5 | 50.0% | 12 | 100.0% | 12 | 85.7% |
|  | No | 7 | 19.4% | 5 | 50.0% | 0 | 0.0% | 2 | 14.3% |
| **24.1. Always wash your boots after working (Choose One)** | Yes | 26 | 89.7% | 3 | 60.0% | 12 | 100.0% | 11 | 91.7% |
|  | No | 3 | 10.3% | 2 | 40.0% | 0 | 0.0% | 1 | 8.3% |
| **25. Always wear rubber gloves on both hands while working (Choose One)** | No | 20 | 55.6% | 5 | 50.0% | 7 | 58.3% | 8 | 57.1% |
|  | Yes | 16 | 44.4% | 5 | 50.0% | 5 | 41.7% | 6 | 42.9% |
| **25.1. Always dispose of the rubber gloves after working (Choose One)** | Yes | 12 | 75.0% | 5 | 100.0% | 5 | 100.0% | 2 | 33.3% |
|  | No | 4 | 25.0% | 0 | 0.0% | 0 | 0.0% | 4 | 66.7% |
| **26. Always wash hands after working (Choose One)** | Yes | 29 | 80.6% | 8 | 80.0% | 9 | 75.0% | 12 | 85.7% |
|  | No | 7 | 19.4% | 2 | 20.0% | 3 | 25.0% | 2 | 14.3% |
| **27. Always remove and wash clothes/aprons after working (Choose One)** | Yes | 28 | 77.8% | 8 | 80.0% | 12 | 100.0% | 8 | 57.1% |
|  | No | 8 | 22.2% | 2 | 20.0% | 0 | 0.0% | 6 | 42.9% |
